# *Xenopus* Meiotic Microtubule-Associated Interactome

**DOI:** 10.1371/journal.pone.0009248

**Published:** 2010-02-17

**Authors:** Vincent Gache, Patrice Waridel, Christof Winter, Aurelie Juhem, Michael Schroeder, Andrej Shevchenko, Andrei V. Popov

**Affiliations:** 1 Inserm Unit 366, DRDC/CS, CEA-Grenoble, Grenoble, France; 2 Max Planck Institute of Molecular Cell Biology and Genetics, Dresden, Germany; 3 Biotechnology Center, Dresden University of Technology, Dresden, Germany; Dresden University of Technology, Germany

## Abstract

In metazoan oocytes the assembly of a microtubule-based spindle depends on the activity of a large number of accessory non-tubulin proteins, many of which remain unknown. In this work we isolated the microtubule-bound proteins from *Xenopus* eggs. Using mass spectrometry we identified 318 proteins, only 43 of which are known to bind microtubules. To integrate our results, we compiled for the first time a network of the meiotic microtubule-related interactome. The map reveals numerous interactions between spindle microtubules and the newly identified non-tubulin spindle components and highlights proteins absent from the mitotic spindle proteome. To validate newly identified spindle components, we expressed as GFP-fusions nine proteins identified by us and for first time demonstrated that Mgc68500, Loc398535, Nif3l1bp1/THOC7, LSM14A/RAP55A, TSGA14/CEP41, Mgc80361 and Mgc81475 are associated with spindles in egg extracts or in somatic cells. Furthermore, we showed that transfection of HeLa cells with siRNAs, corresponding to the human orthologue of Mgc81475 dramatically perturbs spindle formation in HeLa cells. These results show that our approach to the identification of the *Xenopus* microtubule-associated proteome yielded *bona fide* factors with a role in spindle assembly.

## Introduction

Fidelity of spindle assembly and function is of vital importance for the fate of resulting daughter cells. In mitosis, improper chromosome segregation often leads to cell death through mitotic catastrophe [1] and has also been linked to cancer through formation of aneuploid cells [2], [3]. Anomalies during meiotic divisions of the oocyte lead to embryo aneuploidy, and can result in embryonic death or genetic disorders [4], [5], [6] such as trisomies. The latter are often associated with tumor development [7]. Several factors might lead to oocyte-related aneuploidy. They include chromosome non-disjunction in meiosis I and II (MI and MII) [8], as well as diverse factors leading to spindle morphology abnormalities. Such factors may be of a toxic chemical nature [9] or age-related [10], (rev. in [5], [11]). The fact that perturbing functions of proteins other than DNA recombination factors and tubulin may lead to aneuploidy was illustrated by the interference with mitotic kinesin Eg5 in mouse oocytes [12]. Although the relative contribution of the microtubule-dependent spindle abnormalities versus chromosome recombination defects to the total number of embryonic aneuploidy and failures of development events is unclear, evidence suggests that spindle assembly defects account for at least some of them [13].

Although meiosis and mitosis share a lot of similarities, numerous differences exist between the spindles in these types of cells and some of these differences have been already identified: (i) centrosomes are present in somatic cells and absent in oocytes; (ii) kinetochores microtubules appear very late in MI [14]; (iii) Ran GTPase, crucial for spindle assembly in mitosis and MII, seems to be dispensable for MI [15] .

Uncovering novel components of the meiotic microtubule proteome (MeMP) should not only provide clues to pathways common to both mitotic and meiotic spindle, but may reveal factors specific to microtubule-related processes in meiosis.

The spindle is often described as a dynamic assembly of chromosomes, microtubules and regulatory proteins [16]. Both mitotic and meiotic spindle microtubules are stabilized and organized into a bipolar shape by a number of accessory proteins, called microtubule motors and MAPs (for “Microtubule-Associated Proteins”) [17] [18]. Motors, which include cytoplasmic dynein and members of the kinesin superfamily, use the energy of ATP hydrolysis to generate force to move along microtubules (rev. in [19]. Other activities of specialized kinesin-like proteins, such as microtubule depolymerization by proteins of the kinesin 13 family, also require ATP hydrolysis [20]. Major functions of motors in the meiotic spindle include connecting the plus ends of microtubules, positioning the chromosomes at the equator of the spindle and focusing the poles, which is particularly important in the absence of centrosomes. MAPs are usually defined as proteins which bind *in vivo* and *in vitro* to microtubules, co-localize with microtubules in the cell [21] and affect microtubule polymerization dynamics [22], [23]. During oogenesis, MAPs are believed to stabilize microtubules preferentially close to condensed chromosomes, favoring spindle assembly around DNA. Both MAPs and motors can tether other proteins and organelles to microtubules *in vivo* [24], [25]. *In vitro*, the association of motors with microtubules is ATP-sensitive, while MAPs can be eluted by salt. In this way, individual proteins and large protein complexes can be purified on microtubules in amounts sufficient for biochemical analysis of the microtubule-associated proteome [26].

Why use *Xenopus* eggs to study MeMP? Several recent proteomic studies targeted proteins associated with the microtubule cytoskeleton: centrosome from human lymphoblastic cell line KE37 [27], mitotic spindle from HeLa cells [28], midbody in hamster CHO cells [29], and microtubule asters in extracts from mitotic HeLa cells [30]. Using mammalian oocytes to study MeMP would be problematic for availability reasons. On the contrary, *Xenopus laevis* oocytes are easily procured in large quantities and have been long used to study meiosis thanks to the conservation of spindle assembly pathways between vertebrate species. Extracts prepared from unfertilized *Xenopus* eggs [31] represent an abundant source of cytoskeletal and cell cycle proteins. Indeed, during the first 12 divisions after fertilization very little protein translation occurs, with the egg providing most of the proteins needed for these rapid divisions [32]. The uniqueness of *Xenopus* egg extract experimental system lies in the possibility to study many aspects of the spindle assembly in a cell-membrane-free context. Because of this, numerous spindle-related studies have been realized in this system and both the methodology and tools are available for the investigation of novel spindle components. The extracts are usually prepared from eggs arrested in the metaphase of MII (“cytostatic factor-arrested”) and are immediately competent for spindle assembly experiments. We therefore chose *Xenopus* egg extracts as a source of meiotic proteins.

In a recent proof-of-the-principle study we used mass spectrometry and sequence-similarity searches to identify a subset of 41 components of the *Xenopus* MeMP [26]. Building up on these results we set out to decipher the first comprehensive MeMP. Using similar purification strategy together with LC-MS/MS analysis, combined with conventional (Mascot) and sequence-similarity searches [33] we established a catalogue of the proteins bound to microtubules in *Xenopus* egg extracts comprising 318 individual entries. Many of these proteins were either previously uncharacterized or their association with meiotic/mitotic spindle was not reported.

We next assembled the identified proteins into the first literature-curated network of the Meiotic Microtubule-associated Protein Interaction Network (MeMPIN), to show interactions of proteins complexes and individual proteins with microtubules. The network also highlighted a number of proteins and their complexes absent from the mitotic spindle proteome (MSP), suggesting that some of them may have a specific microtubule-related role during oogenesis. Furthermore, we compiled alternative networks, which include high and low-confidence data from interaction databases and yeast-two-hybrid studies on functional and structural orthologues of *Xenopus* proteins. All three Cytoscape-based versions of MeMPIN are interactive and available online.

Further, we evaluated the localization of nine proteins, all of which were not previously reported as components of MSP. For this, we used expression of their GFP-tagged cDNA in cultured cells and addition of GFP-tagged proteins to egg extracts. In this way we showed that seven out of nine new proteins (Mgc68500, Loc398535, Nif3l1bp1/THOC7, TSGA14/CEP41, LSM14A/RAP55, Mgc80361 and Mgc81475) localize to the spindle. Probing the functional role of these proteins, we used siRNA to interfere with the expression of their human orthologues in HeLa cells and showed that depletion of huMGC81475 destabilizes spindle assembly, arresting cells in mitosis.

## Results

### Isolation and Mass Spectrometric Characterization of the Microtubule-Binding Proteome

**Figure 1:**
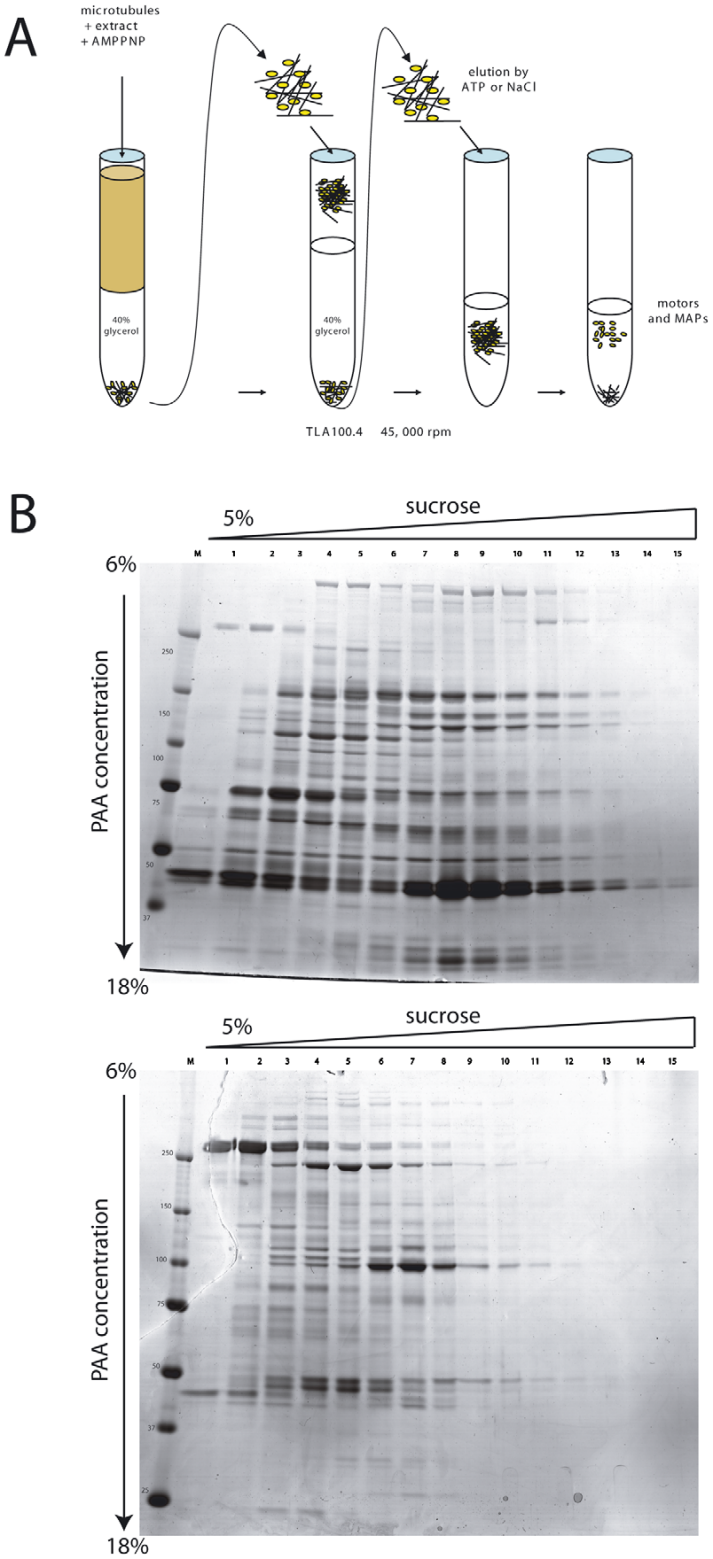
Isolation of microtubule-binding proteins from *Xenopus* egg extracts. **A.** Scheme of the protein purification using precipitation on Taxol-stabilized microtubules. **B.** Coomassie-stained SDS gradient gels showing protein fractions resolved on 5–40% sucrose gradients. Upper and lower gels show the ATP and NaCl elution fractions, respectively. Only fractions 1–15 are shown from the total of 20. PAA- polyacrylamide. kD- kilodaltons.

To isolate proteins associated with microtubules in meiotic egg extracts we followed the protocol described in [26] and [34]. The purification procedure is outlined in Figure 1A. Taxol-stabilized microtubules were added to clarified egg extracts and incubated at 20°C. After several successive centrifugations through a glycerol cushion to remove unattached proteins, microtubule-bound proteins were eluted, first, by 20 mM ATP, and second, by 0.5 M NaCl. The first elution step released all proteins whose association with microtubules was ATP-sensitive (for example, motor proteins), while the second, salt elution step, removed the remaining proteins. To establish the specificity of purification of ATP-sensitive proteins, we omitted AMP-PNP, adding instead to the extract an excess of ATP. This step prevented virtually all of the proteins otherwise recovered in the ATP-fraction from binding to microtubules (Figure S1), proving the veracity of the interaction.

To increase the dynamic range of protein identifications we pre-separated the recovered proteins by one-dimensional (1D) gel electrophoresis. The separation was not sufficient to produce clearly distinguishable patterns, such that the composition and relative abundance of, for example, ATP and salt-eluted protein mixtures could be compared. At the same time, using 2D gels did not allow resolving proteins larger than ∼100 kD at the first dimension (Figure S2). This feature made 2D gels alone not sufficient for our study, as most proteins known to associate directly with microtubule cytoskeleton are rather large molecules. In order to better discriminate between individual proteins prior to the identification by mass spectrometry, we separated the eluted proteins on 5–40% sucrose gradients. After centrifugation, we collected 20 fractions, which were further separated on 1D SDS PAGE gradient gels.

Figure 1B shows Coomassie-stained gels of ATP- and NaCl- eluted sucrose gradients fractions. Only fifteen first fractions are shown as practically no protein bands were observed in later fractions and in the pellet. The majority of proteins were eluted by ATP, rather than by salt. Taxol-stabilized microtubules were quite stable under the conditions used, with relatively little tubulin (bands ca. 50 kD) appearing on both gels. Another interesting feature is that most of the proteins appearing in the high-percentage sucrose fractions (8–13) were found preferentially in the ATP fraction. Such proteins often belong to large protein complexes, such as the multi-ARS complex [26].

ATP- and NaCl- eluted proteins (Figure 1B) were subjected to mass spectrometric characterization. From these two gels we excised 100 bands (60 and 40 from gels with proteins eluted by ATP and NaCl, respectively), corresponding to the apparent sucrose gradient peak of proteins. Moreover, 50 low- and middle-molecular weight protein spots shown on the 2D gels (Figure S2) were additionally used for protein identification. Since each band was expected to be a protein mixture, the excised bands were in-gel digested with trypsin and recovered peptides sequenced by LC-MS/MS.

Altogether, we identified 318 individual proteins from 150 bands/spots (Table S1 contains the full list of identified proteins and indicates their provenance) from which 43 were known MAPs, microtubule motors and related proteins, previously reported in association with microtubule cytoskeleton (Table S2, columns “D” and “J”). According to the published evidence, the above proteins or their previously characterized orthologs from other species localized to interphase and/or spindle microtubules, kinetochores, midbody and the centrosome.

We next grouped all identified proteins into fourteen functional categories (Figure S3 and Table S1). Many proteins were found both in ATP- and NaCl-eluted fractions, some of them possibly due to the sequential elution first by ATP and second by NaCl. Incomplete elution by ATP is common in experiments where AMPPNP is used to enrich motor proteins in the microtubule pellet. Nonetheless, in the ATP fraction we observed many more proteins from “signaling”, “metabolism”, “RNA transcription and processing”, “protein synthesis and folding” and ”vesicular traffic” categories. Unexpectedly, most MAPs (XMAP215, MAP4, p150^Glued^, EB1, Lis1, CLIP170, etc.) were not only eluted by salt, but also by ATP. This suggests that at least a fraction of these proteins were recruited to microtubules in ATP-sensitive complexes, perhaps via the association with dynein or other motor proteins. Similarly, some motors were found in the NaCl fraction and although the sheer number of motors found in the ATP and NaCl fractions is comparable (10 and 8 respectively), only 3 motors were found in both fractions. This suggests that motors found exclusively in the NaCl fraction (XKid, KIF3A, XKCM1, XKlp3 and XCTK2) were retained on microtubules via an ATPase-independent domain [35] or via interaction with other microtubule-binding proteins.

A few centrosomal proteins (γ-tubulin, NEDD1, TSGA14/CEP41, GCP2) were also identified. Although centrosomes are absent in oocytes, this finding is not surprising, as many centrosomal proteins interact (directly or indirectly) with microtubules in oocytes and egg extracts. For example, γ-tubulin was found not only at the centrosomes, but also at the microtubules of mitotic spindles and on spindles assembled in egg extract [36], [37]. Another centrosomal protein found in our screen, NEDD1, co-precipitates with γ-tubulin complex (γ-TURC) and binds to microtubules [38].

Despite of our use of cytochalasin B in extract preparation to prevent a massive contamination of the microtubule pellet with polymerizing actin filaments [39], we identified a number of actin-related proteins. Additionally, we found a number of proteins known to interact with both actin and microtubules, such as MACF1 and plectin, pointing to a strong link between these two types of filaments in egg extract system. Indeed, the actin cytoskeleton plays an important role in cell division processes: in cytokinesis it forms a contractile ring and in oocytes during MI (at least in starfish experimental model) actin network congression brings chromosomes together to the point where the spindle forms [40]. Moreover, myosin-10 integrates the F-actin and microtubules both in meiosis and mitosis [41], [42].

We also identified some ribosomal and RNA-related proteins, and a large number of DNA-related proteins, which mostly fall into the categories of structural maintenance of chromosomes, DNA transcription and replication. Some of the known spindle components, like NUMA, were not found in our preparations, most likely because of the stringent purification procedure that we adopted. Indeed, the “extract clarification” centrifugation step allows avoiding contamination with other than microtubules macromolecular complexes and organelles, but at the same time removes large protein complexes, such as known to exist for proteins like NUMA [43].

In conclusion, we isolated 318 individual proteins, among which are most of the proteins currently known to localize to microtubules and/or spindles assembled in *Xenopus* egg extracts. This result suggested that other proteins and protein complexes identified in both ATP- and NaCl-eluted fractions are also specifically bound to microtubules and represented previously unknown spindle components.

### Building a Network of the Microtubule-Associated Interactome in Meiosis

**Figure 2:**
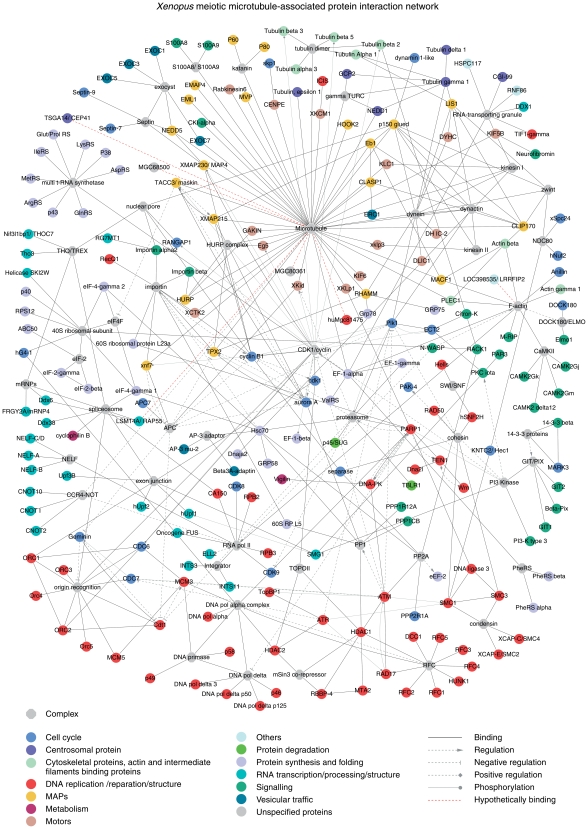
Meiotic microtubule-associated protein interaction network. Map contains 218 proteins, 75% of all characterized proteins identified in our study, and 51 complexes. Note that EF1-α, CKI-α, Need1, tubulin ε and Plectin 1 are shown as MAPs because they can bind to microtubules directly. The identified by us spindle components (Mgc68500, TSGA14/CEP41, LSM14A/RAP55A, Mgc80361, Mgc81475; see Figure 3) are linked to the microtubule using a dashed line, since their binding may be indirect. Similarly, a dashed line is used to show the interaction of Loc398535/LRRFIP2 with actin.

Recent advances in bioinformatics, data and text mining allow integrating experimental data in the form of user-defined graphical networks. Such models represent a snapshot of a particular biological interaction or cellular process and help systems-level understanding of the organization and regulation of complex protein assemblies. To evaluate functional connections of proteins with microtubules and with each other, we created a network of MeMP. We first used all 290 identified known proteins (a total of 318 proteins minus 28 uncharacterized ORFs) to find all direct links with the microtubule cytoskeleton. As a result, we compiled a Core map containing 50 proteins (tubulins and generally accepted MAPs and motors) in direct binding relations, previously observed either in somatic cells or in oocytes (Figure S4). Second, we related all identified known proteins between themselves and fused the resulting assembly with the Core map to obtain a network of 218 proteins (75% of the identified annotated proteins) and 51 complexes with 440 functional interactions (Figure 2). All entries and references pertaining to their links are listed in Table S4.

We then compiled the layout of the network in Cytoscape, an open source software platform for visualizing molecular interaction networks [44]. Within the Cytoscape session file, we provide links to UniProt, GeneCards, BioGrid and Entrez databases for each protein, and PubMed literature references for each protein relationship or protein complexes (http://www.biotec.tu-dresden.de/schroeder/mempin/start.jnlp); file S1 MeMPIN.cys, for Meiosis Microtubule-related Proteins Interaction Network). Using bioinformatics tools, we further probed existing protein-protein interaction databases (BIND, DIP, HPRD, IntAct, MINT, and NetPro) and identified a number of high- and low-confidence binary interactions, which we added to the curated MeMPIN. The resulting networks (files S2 and S3), show 226 and 231 proteins, and include 518 and 590 functional links, respectively.

We deliberately refrained from merging the identified proteins into complexes specific for the kinetochores and the centrosome, since we found in our screen only a limited number of proteins related to these structures. The absence of most of kinetochore proteins is not surprising, as chromosomes should have sedimented during the extract clarification step. Likewise, the centrosome is absent in oocytes and arrives with the sperm cell during fertilization. A few of the centrosomal proteins that we identified are in fact also found on microtubules in meiosis and on interphase microtubules in somatic cells (see above on γ-tubulin and TSGA14). Some of these proteins are known spindle components, however their direct binding to microtubules was not demonstrated before.

### Validation of Novel Spindle Components

One of the motivations for this work was to identify novel spindle components. To this goal we selected nine proteins, all of which were uncharacterized or poorly studied proteins and absent from MSP. To test if these proteins were a part of the spindle in meiosis, we added their bacterially produced GFP-fusion versions to meiotic egg extracts. Similarly, to test if these proteins were authentic components of the spindle in somatic cells, we expressed their cDNAs as GFP-fusions in cells. To this end we obtained full-length cDNAs for nine putative spindle components (Table 1; six cDNAs from *X. laevis* and three from *H. sapiens*) and cloned them into both eukaryotic and prokaryotic expression vectors to fuse them at the 5′ of the ORF with an EGFP gene. These constructs were used for production of recombinant proteins in *E.coli* or transfected into *Xenopus* or human (where appropriate, for human cDNAs) cells.

**Table 1:** Summary data on nine candidate proteins with their localizations.

No	Clone name/ ExPASy Acc. No	Spe- cies	Other names and references	Predicted MW, kD	Domain(s)	GFP-tagged protein in *Xenopus* cells	GFP-tagged protein in human cells	GFP-tagged protein in egg extracts
1	Nif3l1bp1/ Q7SZ78	XL	THOC7 ^1^	23.4	2 x coiled coil	Cytoplasme, spindle	NA	Spindle microtubules, poles
2	Mgc80835/ Q6GNW0	XL	LRRFIP2 ^2^	46.2	3 x coiled coil	Actin, cell-cell junctions	NA	-
3	Mgc68500/ Q6PB22	XL	Similar to IHABP4 ^3^	45.5	HABP4_PAI-RBP1	Cytoplasme, spindle, mitotic chromosomes	NA	Spindle microtubules
4	Loc398535/ Q6GQL3	XL	unstudied	38.7	BAT2	Cytoplasme, spindle	NA	Weak signal on spindle microtubules
5	Mgc80361/ Q6AXA1	XL	unstudied	42.8	–	Nucleus	NA	Spindle microtubules
6	Mgc81475/ Q6NRT3	XL	Smu1 ^4^	57.6	7 x WD LisH CTLH	Cytoplasme, nucleus midbody	NA	Spindle microtubules, poles
7	TSGA14/ Q9BYV8	HS	Cep41 ^5^	41.4	Rhodanese_3	NA	Interphase microtubules, centrosome	Spindle microtubules
8	Kiaa1799/ Q96B95	HS	unstudied	71.9	3x EFHand	NA	Cytoplasme	–
9	LSM14A/ Q8ND56	HS	RAP55A ^6^	50.5	–	NA	P-bodies microtubules, spindle, midbody	Spindle microtubules

Localization of GFP-tagged *Xenopus* and human candidate proteins was observed in live A6 and HeLa cells, respectively. NA – not applicable. ExPASy - ExPASy Proteomics Server (http://www.expasy.org). LisH - Lissencephaly type-1-like homology motif. CTLH - C-terminal to LisH motif. EFhand - calcium-binding domain – found in calcium-binding proteins. References: 1- [70]; 2- [45]; 3-[68]; 4- [46]; 5-[27]; 6-RAP55 [72].

**Figure 3:**
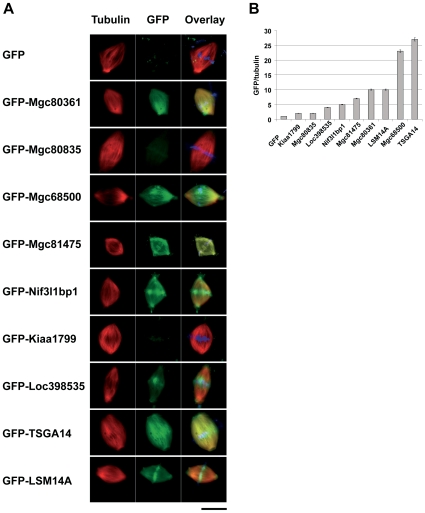
Localization of GFP-tagged candidate proteins at egg extract spindles. **A.** Spindles assembled in egg extracts in the presence of GFP-tagged candidate proteins. Size bar – 20 µm. Overlay column shows red (tubulin), green (GFP) and blue (DNA) signals. **B**. Relative intensities of the GFP fluorescence of the spindles assembled in the presence of nine GFP-tagged candidate proteins, normalized to the Rhodamine-tubulin signal. Values represent average from at least 10 spindles per sample. The ratio of GFP signal to tubulin fluorescence intensity for GFP alone was considered as 1. Error bars show s.e.m.

Figures 3A shows spindles assembled in egg extracts in the presence of GFP-tagged candidate proteins and results are summarized in Table 1. We found that seven out of nine proteins tested clearly localized to spindles. We next quantified the amounts of GFP-tagged candidate proteins on the spindles (normalized to the tubulin signal), providing a measure of the proteins' relative abundance on spindle microtubules (Figure 3B). Interestingly, two proteins - GFP-Loc398535 and GFP-TSGA14 - affected the morphology of the assembled spindles. Thus, although GFP-Loc398535 staining on extract spindles was relatively weak, the protein's addition to egg extracts resulted in the formation of spindles with reduced microtubule density (average spindle microtubule signal 41.7%±9 versus 100%±18.6 in control). This result suggests that the overexpression of GFP-Loc398535 interferes with microtubule polymerization or reduces their bundling. An opposite effect was observed in the case of GFP-TSGA14, whose addition resulted in the formation of large spindles (average spindle microtubule signal 157.3%±14.9).

We next verified the localization of the candidate proteins in somatic cells. Figure S5 shows the localizations of the GFP-tagged proteins in live cells. We found that four (Mgc68500, Loc398535, Nif3l1bp1 and LSM14A) candidate spindle components clearly localized to the spindle in live cells (Figure S5B). GFP-TSGA14 was observed on microtubules and centrosomes in live and fixed interphase cells (Figure S5C and Figure S8). Moreover, we observed GFP-LSM14A on midbody and its overexpression induced a pronounced microtubule bundling in interphase cells, frequently observed with proteins directly binding to microtubules (Figure S5D).

Mgc80835 was recently termed leucine-rich repeat flightless-interacting protein 2 (LRRFIP2) and found to activate the canonical Wnt signaling pathway upstream of ctnnb1/beta-catenin [45]. Interestingly, we found that GFP-tagged Mgc80835, which is weakly similar to myosin heavy chain, clearly localized to: (i) actin stress fibers, which may explain its appearance in our preparation (the role of actin in oocytes is commented on above); (ii) cell-cell junctions (not shown), suggesting a role in cell signaling or membrane transport. Kiaa1799 is a hypothetical protein, which we found in the cytoplasm. Mgc81475 is the *Xenopus* orthologue of the *C. elegans* protein Smu-1 suppressor of mec-8 and unc-52, which is believed to regulate the alternative splicing of pre-mRNAs [46] and was found by us in the nucleus and midbody. Mgc80361 (similar to human FAM98A) is an unstudied protein, which in somatic cells localized to the nucleus in interphase cells and was diffuse in the cytoplasm of mitotic cells.

Summing up, expression in cells or addition to egg extracts of nine GFP-tagged proteins allowed us to identify at least seven novel spindle components.

### Functional Evaluation of Six Candidate Proteins Implicates Mgc81475 in Spindle Formation

To explore the role of the candidate proteins in spindle formation we depleted their closest orthologues in HeLa cells using two different siRNA for each gene (Table S6). Cells were fixed 72 hours after transfection and stained with anti-tubulin and anti-phospho histone 3 (H3P) antibodies to visualize mitotic cells.

We found that the effect of RNAi on the mitotic index was most prominent in the case of the two siRNAs directed against the human orthologue of Mgc81475 (huMgc81475). Depleting cells of huMgc81475 resulted in mitotic arrest (mitotic index was 7.54 and 6.9, for siRNA#3 and siRNA#4, respectively; versus 4.3 – average for ten other siRNAs used in parallel, see Figure S6). In another experiment, we transfected HeLa cells with the mixture of siRNA#3 and siRNA#4 and registered at 48h the mitotic index of 8.1±1.35 versus 3.8±0.67 in control (N = 3108 and 3476, correspondingly). Of note, in samples transfected with siRNA#3 or siRNA#4 we observed a large number of dead cells (not shown), which could explain why the effect of the RNAi on the mitotic index was relatively modest.

**Figure 4:**
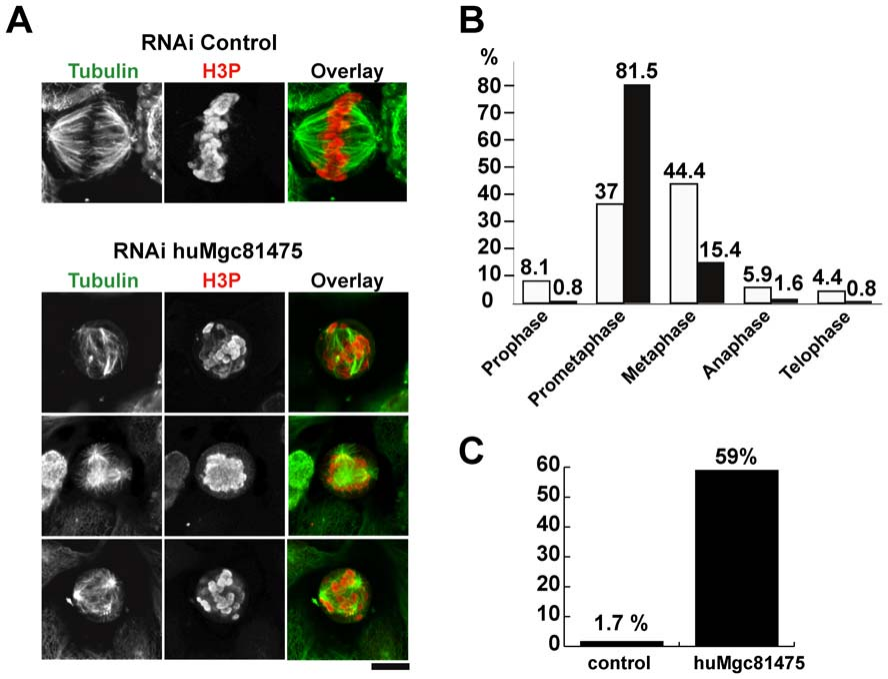
huMgc81475 depletion perturbs spindle assembly. **A.** Immunofluorescence on fixed HeLa cells after transfection with control and huMgc81475 siRNAs. Cells were stained with anti-phospho-histone H3 (H3P) and anti-tubulin antibodies. Bottom panel shows three representative phenotypes in cells treated with huMgc81475 siRNAs #3 and 4. Size bar – 10 µm. **B**. Quantification of phenotypes observed as a result of the huMgc81475 knockdown. Morphological stages of mitosis were evaluated for control (white bars; N = 135) and huMgc81475 siRNA-treated H3P-positive cells (black bars; N = 254). **C**. Percent of mitotic cells with unaligned chromosomes in control- and huMgc81475 siRNA-treated cells. Only mitotic cells with bipolar structures were evaluated.

Confocal microscopy on fixed cells treated separately (data not shown) with either siRNA#3 or siRNA#4 showed identical defects in spindle formation, while simultaneous transfection with siRNA#3 and siRNA#4 had synergistic effect (Figure 4A). In siRNA-treated cells we observed bi- or multipolar structures with reduced density of microtubules, multipolar spindles and microtubule asters. Quantification of the observed phenotypes (Figure 4B) suggests that cells arrest before metaphase, which is compatible with mitotic arrest due to microtubule destabilization. Interestingly, although in siRNA-treated samples the percentage of cells in metaphase decreased dramatically (15.4% versus 44.4% in the control), those which were found in metaphase (or in metaphase-like state with well-separated poles) presented unaligned chromosomes (59% versus 1.7% in the control; Figure 4C).

These data suggest that huMgc81475 may play a role in the microtubule stabilization in mitosis.

## Discussion

### Microtubule-Associated Proteome in Meiosis

Numerous recent proteomic studies aimed the accurate characterization of proteomes related to the microtubule cytoskeleton. At the same time, the identification of MeMP was hampered by the availability of sufficient material and by the limitations of bioinformatics tools. Short of using mammalian oocytes, research relied instead on model organisms, such as *Xenopus,* where a large amount of material is easily available.

Three major limitations are usually considered in the interpretation of functional proteomics datasets - (i) fidelity of the protein identification; (ii) proteins missed; and (iii) “contaminating” proteins found.

- Using *Xenopus* as a model system for proteomics studies used to be problematic until lately as the genome of this tetraploid species is yet to be fully sequenced. However, recently a large number of *X. laevis* and *X. tropicalis* (a member of the same genus closely related to X. laevis) cDNAs, ESTs, genomic sequences have become available. Moreover, formidable advances in sequence similarity searches make it possible to identify with a high fidelity proteins whose exact sequence is missing in the database, using their likeness to orthologues from other species.
- Certain fraction of proteins may go undetected in any large-scale proteomics project. To make sure that we did not miss a large proportion of the microtubule-associated proteome, after the identification of 318 proteins from 150 samples we analyzed by mass-spectrometry 22 randomly chosen discrete minor protein bands (from gels shown in Figure1B). In this second round of analysis we were unable to identify any additional proteins, suggesting that we detected most of proteins isolated on microtubules. The fact that the above mentioned 22 bands contained only already found in other samples proteins is not surprising, as many cytoskeletal proteins are regulated by phosphorylation and, isolated from meiotic/mitotic cells, migrate as several bands of different apparent molecular weight.
- Affinity purification of protein complexes from cellular extracts is often accompanied by co-isolation of “background” proteins. To minimize this effect, we clarified egg extract prior to microtubules addition. We also controlled the purity of the preparation by adding an excess of ATP to extracts, which prevented virtually all of the proteins otherwise recovered in the ATP-fraction from binding to microtubules (Figure S1). This stringency obviously comes at the price of missing proteins that localize to microtubules exclusively as members of large complexes or make part of organelles and could be thus removed by extract clarification (see above on NUMA).

### Meiosis Microtubule-Associated Protein Interaction Network

Microtubules are at the heart of the complex cell division machinery, which ensures equal distribution of daughter chromosomes, organelles and cytoplasm. It is therefore not surprising that hundreds of proteins are associated with microtubules either directly, or via organelles such as centrosomes [27], chromosomes [47] or ribosomes [26], [48].

To integrate our results, we built a network of MeMP. Such a network of a specialized proteome is particularly credible when its components are isolated by virtue of their physical interaction. Unlike the yeast two-hybrid system [49], which is a powerful method for mapping binary interactions, isolation of proteins by virtue of their physical interaction with microtubules is bound to yield multi-protein complexes, thus increasing the interactome complexity. In the centre of the protein interaction network (Figure 2) are proteins interacting directly with microtubules (Core map, Figure S4).

Core map reveals that the majority of known MAPs and motors interact mostly with microtubules, with a small number of them showing also binary interactions (binding) between each other. Such are interactions of XMAP215/TACC3, XMAP215/EB1 or the recently described HURP/XMAP215/Eg5/TPX2 complex, although the exact hierarchy of the latter complex is unclear [50]. Interestingly, most of the MAPs connected to each other are functionally and spatially related to the microtubule plus ends: Lis1, EB1, dynein heavy chain (DNCH1), p150^Glued^, Clip170, CLASP1 and XMAP215, highlighting the complexity of interactions between MAPs at the tips of growing microtubules [51]. Cytoplasmic dynein complex is also connected with a large number of proteins known to depend on it for the minus-end directed transport. A number of relatively little studied MAPs such as MVP [52], EXOC7 [53] and HOOK2 [54] are also shown as binding directly to microtubules.

Multi-aminoacyl-tRNA synthetase complex (multi-ARS complex) was repeatedly found in association with microtubules in meiosis [26] and mitosis [28] and could bind microtubules through association with either EF-1α or ribosomes [48], or via another yet unknown partner(s). We also show a complex termed “RNA-containing granules” after we found a number of proteins making part of these granules, which are transported on microtubules by kinesin I [55].

Apart from the generally known spindle components such as dynein/dynectin and tubulins, several other “hubs” are connected with a large number of entries. These are: importin (12), CDK/cyclin (22), F-actin (12), PARP1 (13), ATM (11), ATR (6), proteasome (14), with the number of nearest connecting neighbors for the hub shown in parenthesis. Interestingly, most of the ribosomal proteins found in our study belong to the small, 40S ribosomal subunit, which is known to associate with another multiprotein factor that we have identified, the cap-binding complex eIF4F [56]. Finally, the map shows multiple complexes implicated in RNA and DNA transcription, DNA replication and structure maintenance.

**Figure 5:**
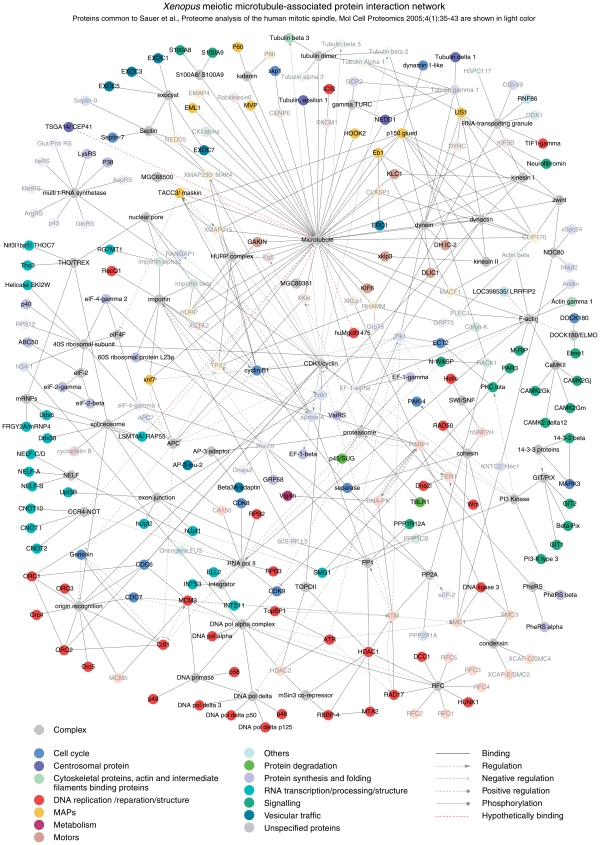
Proteins common to meiosis microtubule-associated protein interactome and mitotic spindle proteome. Same as Figure 2 with proteins common to both MeMP and MSP shown in light color. Note that some of the 90 common proteins are not shown on the network (see Table S3 legend).

Comparing all the identified *Xenopus* proteins to previously reported components of the human MSP [28], we used reciprocal BLAST searches to relate the orthologues. Interestingly, we found a relatively modest overlap between the two proteomes - they only share 90 entries (28% out of 318; Figure S7; Table S3). Among known spindle components we identified 11 MAPs (plus tubulins δ1 and ε) and 3 motors (plus dynein light intermediate chain 1 and kinesin light chain), that were not reported by [28] (Table S2). Similarly, in MeMP we did not find a number of proteins present in MSP, which could be partially due to the different purification strategies. Probing the network for pathways that may be specific for MeMP we modified Figure 2 to highlight proteins absent from MSP (Figure 5). In difference with MSP, in meiotic microtubule-related interactome we observed a number of proteins comprised within and/or interacting with the following complexes: origin recognition complex; DNA pol δ and α; RNA pol II and CCR4-NOT transcriptional complex; exon junction; CaMKII. While the presence of DNA polymerases in microtubule preparations is usually assumed to result from association of egg chromosomes with microtubules, our map suggests an alternative link: these complexes may be connected to microtubules via PARP1, which is a part of a multiprotein DNA replication complex [57]. Interestingly, the complexes of replication factor C (RFC), cohesin (SMC1/SMC3) and condensin (SMC2/SMC4) are found both in MeMP and MSP, while the third SMC-family complex, DNA repair complex SMC5/SMC6, is only found in MeMP (not shown on the network in Figure 2), which may reflect the importance of this complex in meiotic recombination.

Furthermore, we found a number of proteins involved in the post-transcriptional events: these proteins are shown on the Network as part of the following complexes: spliceosome, mRNPs, exon junction and THO/TREX. Most of these proteins are absent from MSP and their main functions in oocytes relate to mRNA processing, storage and translational repression. These proteins dynamically interact with mRNA and with each other and some of them are known to participate in more than one type of the ribonucleoprotein complexes [58], [59], [60].

An ubiquitin ligase Xnf7, which inhibits APC in egg extracts [61] and contributes to the stability of egg extract spindles, is abundant on extract spindles. At the same time, Xnf7 is only weakly noticeable on the poles of mitotic spindles [62], and thus could be a meiosis-specific spindle factor. In line with this reasoning, the closest human orthologue of Xnf7 - TRIM69 (42% identity to Xnf7) - was not reported for MSP [28]. Another complex found by us, the exocyst (and its component EXCO7), is missing in MSP, although it was observed on mitotic spindles by immunofluorescence and is required for the completion of cytokinesis [63]. Lis1 and EB1 are two important MAPs which are present on microtubules plus ends, were also found by us and missing from MSP, most likely because of different purification strategies employed.

Although dynamic properties are usually excluded from complex protein-protein interaction networks [64], many of the interactions depicted in Figure 2 are in fact spatially and temporally regulated and only happen in the cell in the right place and at the right time. Therefore, the protein interaction network should be regarded as a convenient graphic tool enabling quick access to complex, often indirect, and not-so-obvious possible relationships between microtubules, individual proteins and their complexes.

We propose that such networks may further evolve to describe not only MeMP, but also the mitotic spindle of metazoan organisms with its much more complex apparatus, including kinetochores, centrosomes and chromosomes. Indeed, the respective proteomes of the mitotic spindle [28], centrosomes [27] and mitotic chromosomes [47] have recently been elucidated, paving the way towards integration of these data. Moreover, future challenges lies with making such networks dynamic, to provide a measure of the quantitative and qualitative changes of the interactomes in different stages of the cell cycle and in different cell types.

### Novel Spindle Components

Most of known spindle proteins have an important role in its function. Some of the rare, but notable exceptions include XMAP230 (*Xenopus* MAP4), whose depletion does not significantly perturb spindle formation, at least in the egg extract experimental system [65]. Thus, by virtue of their localization, any newly found spindle components automatically become suspect regulators of the spindle assembly and potential targets for anti-mitotic drugs. Expression of GFP-tagged proteins makes it possible to observe proteins localization in live cells [66] although GFP-tagging may in some cases interfere with the correct localization of expressed proteins [67]. GFP-tagged versions of four novel candidate proteins clearly localized to mitotic spindles in live cells. Adding the same GFP-tagged recombinant proteins to egg extracts, we showed that, excepting GFP-Kiaa1799 and GFP-Mgc80835, all of the nine candidate proteins clearly localized to spindle microtubules.

Structurally and functionally, all nine candidates belong to different classes and were chosen by us because they were unstudied or poorly studied and absent from MSP. Mgc68500's closest orthologue in man is intracellular hyaluronan-binding protein IHBP4 (HABP4) [68], reminding another hyaluronan-related protein, RHAMM, which participates in Ran-dependent spindle assembly [69]. Nif3l1bp1/THOC7 is a part of THO complex, implicated in mitotic recombination and transcription elongation [70]. Interestingly, in MeMP we detected another member of the THO complex (THOC3), while THOC2 was detected in MSP [28], suggesting that THO complex is a part of the spindle machinery.

Coiled-coil containing protein TSGA14 (Cep41) was recently described as a centrosomal component [27]. The fact that we found GFP-tagged TSGA14/Cep41 at the spindle in egg extracts and associated with microtubules in interphase cells suggests that this protein may also be a MAP, as many MAPs have both centrosomal and microtubule localization [71]. We did not observe live mitotic cells expressing GFP-TSGA14, which suggests that the overexpression of GFP-TSGA14 interferes with entry into mitosis.

LSM14A/RAP55A is involved in mRNA storage in interphase cells and represses translation in *Xenopus* oocytes [72]. Although most mRNAs are silent in oocytes, there is evidence that specific mRNAs [60] localize on mitotic and meiotic microtubules, and some of them, coding for key meiotic/mitotic proteins, such as cyclin B1, may be translated in oocytes locally, on or near the spindle [73]. This concept was further underscored by the findings that spindles assembled in *Xenopus* egg extracts are extensively decorated by ribosomes [26]. It is therefore possible that proteins such as LSM14A/RAP55A play a role in the local regulation of translation on the spindle.

Depletion of the six candidate proteins by RNAi allowed us to evaluate their functional role in spindle formation in somatic cells. Knocking-down the human orthologue of Mgc81475 clearly implicated this protein in the microtubule stabilization in mitosis. Interestingly, when expressed in *Xenopus* cells, GFP-Mgc81475 was not observed on the spindles. Instead, when added to egg extracts, GFP-Mgc81475 intensively decorated spindles assembled around sperm nuclei. The lack of the spindle staining in cultured cells may have resulted from the overexpression phenomenon – indeed, the presence of too much of a protein with a tightly regulated concentration may have prevented its correct localization in cells.

Different cell cycle and developmental stages impose specific shapes on the microtubule cytoskeleton. In parallel with the changing role of the microtubule cytoskeleton so may vary the composition of the microtubule-associated proteome. At least two mechanisms are known for spindle assembly – one, in somatic cells, involves centrosomes as major microtubule nucleation centers; another, acentrosomal mechanism, is characteristic of oocytes, devoid of centrosomes (rev. in [15], [74]). Recent evidence also suggests that although in MII spindle assembly depends on the high local concentration of Ran-GTP, in MI spindle forms in the absence of the centrosome and independently of the Ran-GTP pathway [15].

It is also believed that the centrosomal pathway is, at least partially, redundant in somatic cells and in the absence of centrosomes chromosomes alone can drive spindle formation in mitotic somatic cells [75]. Because of such redundancy, it is not surprising that most of the spindle assembly components described so far are localized to the spindle in both somatic cells and oocytes. It seems likely that most of proteins physically present on microtubules of meiotic spindles will be found on mitotic spindles as well. However, the relative abundance of these proteins and/or the weight of their functional contribution to spindle assembly in these two types of spindles may differ significantly. To address the issue, we quantified the binding of GFP-tagged proteins to spindle microtubules in egg extracts, giving us a measure of their relative abundance on the spindles. However, these results should be treated with caution, as both the presence of the GFP-tag and the fact that recombinant proteins were produced in bacteria, could affect the abundance of the proteins on the spindle.

Interestingly, peptides corresponding to the human orthologues of Mgc68500, Loc398535, Nif3l1bp1/THOC7 and LSM14A/RAP55 were not detected in MSP [28]. However, a number of well-known spindle proteins found in our study are also missing from MSP [28], suggesting that quantitative, rather than qualitative differences may be responsible for this discrepancy.

In our discussion we mainly focused on the meiotic/mitotic spindle connections of the identified by us proteins. Nonetheless, it is known that amphibian eggs contain a vast store of proteins, mRNAs, RNPs and other organelles, required for the early embryonic divisions. Many of these components are associated with microtubules and not exclusively during cell division phase of the cell cycle. Therefore, although we isolated proteins from meiotic egg extracts, it is possible that some of them may be involved in microtubule-dependent processes different from spindle assembly.

## Materials and Methods

### Isolation of Microtubule-Binding Proteins from *Xenopus* Egg Extracts

All animal experiments were conducted by trained personnel in accordance with the guidelines of the Grenoble Institute of Neurosciences Ethics Committee. Proteins were purified as described in [34] starting with 8 ml of *Xenopus* egg extracts. Tubulin was purified from bovine brain tissue according to [76]. ATP- and NaCl-elution fractions (0.5 ml) were concentrated to 100 µl using microconcentrators Vivaspin 500 (VS0101; Vivascience) with a 10 kD cut-off. For the 2D analysis the samples were desalted by diluting the concentrated proteins to 0.5 ml with 10% PBS and re-concentrating them as above. Concentrated samples were loaded on the top of a 3.4 ml sucrose gradient in Beckman centrifuge tubes, Cat. No. 349622. To prepare 5-40% sucrose gradient, 1.7 ml of each sucrose solution (5 and 40%; filtered through a 0.22 μm membrane) were mixed using a peristaltic pump and a home-made gradient mixer. Samples were resolved by centrifugation in Kontron TST-54 rotor at 35,000 rpm (100,000g) for 12 hours at 4°C. After centrifugation samples were fractionated by manually collecting 200 µl-probes starting at the top of the gradient. Each probe was analyzed on a 6–20% gradient SDS-PAGE and stained with Coomassie Blue. Experiment in Figure S1 (Lane 3) was carried using recombinant p50/dynamitin (courtesy of T. Wittmann).

### Spindle Assembly in Egg Extracts in the Presence of GFP-Tagged Proteins

CSF (cytostatic factor) - arrested *Xenopus* laevis egg extracts were prepared essentially as described in [39]. Sperm nuclei were prepared as described in [77] and added to extracts to a final concentration of ∼100 nuclei/µl. Cy3B-labelled tubulin at 10 mg/ml was obtained following the protocol of [78] and added to extracts at 1/40 of the final reaction volume (30 µl). His6-tagged non-degradable cyclin B1 of *X. laevis* (unpublished, AP) was added to extracts at 20 µg/ml final concentration. His6-EGFP or his6-EGFP-tagged candidate proteins were added at ∼0.5 µM. Upon spindle assembly after 1 hour incubation at 20°C, samples were fixed by adding 1 ml of 1% GA, 0.1% Triton X-100 in BRB80 buffer (80mM K-PIPES, pH 6.8, 1mM MgCl, and 1mM EGTA, titrated to pH 6.8 with KOH) and immediately centrifuged onto glass coverslips, followed by post-fixation in -20°C anhydrous methanol for 15 min as described in [79]. Coverslips were rinsed in PBS and free aldehyde groups were “quenched” using two consecutive 5 min incubations in 1 mg/ml NaBH_4_ in PBS. DNA was stained with Hoechst 33258 at 2 µg/ml in PBST. After several washes in PBST, coverslips were mounted on glass slides using the mounting liquid Fluorsave™ Reagent (Calbiochem). For indirect immunofluorescence we used the following filter cubes from Omega: XF03 (DNA), XF100-2 (EGFP), and XF102 (Cy3B). To measure the relative intensities of GFP-tagged proteins on the spindles assembled in egg extracts we used the linescan function of Metamorph on 12-bit images taken under identical conditions. Values correspond to the average of at least 10 spindles relative to his6-GFP (1.0). To evaluate the effect of GFP-Loc398535 and GFP-TSGA14 addition on average microtubule mass of the spindles assembled in egg extracts we measured total Rhodamine signal in control (GFP) and after addition of GFP-fused candidate proteins. Values are represented as percentage from control (100%) ± standard deviation (shown in %).

### Protein Identification by Mass Spectrometry and Classification

Protein bands were processed for mass-spectrometry as described in [34]. Briefly, bands were excised and in-gel digested as described in [80]. Dried peptide extracts were re-dissolved in 0.05% (v/v) trifluoroacetic acid (TFA) and loaded using FAMOS autosampler on a nanoLC-MS/MS Ultimate system (Dionex, Amstersdam, The Netherlands) interfaced on-line to a linear ion trap LTQ mass spectrometer (Thermo Fisher Scientific, San Jose, CA). The mobile phase was composed of were 95:5 H_2_O: acetonitrile (v/v) with 0.1% formic acid (solvent A) and 20∶80 H_2_O: acetonitrile (v/v) with 0.1% formic acid (solvent B). Peptides were first loaded onto a trapping microcolumn C18 PepMAP100 (1 mm×300 µm ID, 5 µm, Dionex) at a flow rate of 20 µl/min in 0.05% TFA and then back-flush eluted and separated on a nanocolumn C18 PepMAP100 (15 cm×75 µm ID, 3 µm, LC Packings) at a flow rate of 200 nl/min in the mobile phase gradient: from 5 to 20% of solvent B in 20 min, 20 to 50% B in 16 min, 50 to 100% B in 5 min, 100% B during 10 min, and back to 5% B in 5 min; %B refers to the content of solvent B in A+B mixture. Peptides were infused into the mass spectrometer via a dynamic nanospray probe (Thermo Fisher Scientific) and analyzed in positive mode. A silicatipTM uncoated needle (20 µm ID, 10 µm tip ID) (New Objective, Woburn, MA) was used with a spray voltage of 1.8 kV, and the capillary transfer temperature was set to 200°C. In a typical data-dependent acquisition cycle controlled by Xcalibur 1.4 software (Thermo Fisher Scientific), four most abundant precursor ions detected in the full MS survey scan (m/z range of 350–1500) were isolated within 4.0 amu window and fragmented. MS/MS fragmentation was triggered by a minimum signal threshold of 500 counts and carried out at the relative collision energy of 35%. Spectra were acquired under automatic gain control (AGC) in one microscan for survey spectra and in three microscans for MS/MS spectra, with the maximal ion injection time of 100 ms per microscan. The m/z of fragmented precursor were then dynamically excluded for another 60 seconds. From raw files, MS/MS spectra were exported as dta (text format) files using the script extract_msn.exe (Thermo Fisher Scientific) under the following settings: peptide mass range: 500–3500; minimal total ion intensity threshold: 1000; minimal number of fragment ions: 15; precursor mass tolerance: 1.4 amu; group scan: 1; minimum group count: 1.

For protein identification, dta files representing individual tandem mass spectra were converted into a single mgf- file and submitted to database search using Mascot software version 2.1 (Matrix Science Ltd, London, UK) installed on a local 2 CPU server. Database searching settings: tolerance for precursor and fragment masses were 2.0 and 0.5 Da respectively; instrument profile: ESI-Trap; database: MSDB (updated May 15, 2005); fixed modification: carbamidomethyl (cysteine); variable modification: oxidation (methionine). Protein identified with at least two peptides and a Mascot score exceeding 100 were considered as significant hits. Borderline hits were further validated by *de novo* sequencing and sequence similarity search with MS BLAST. To this end, dta files corresponding to the borderline hits identified by Mascot were retrieved from the mgf file by an in-house script and were interpreted *de novo* using a modified version of PepNovo software [81]. The interpretation of each dta file resulted in several peptide sequence proposals, and all candidate sequences were assembled into a single query for MS BLAST search [82] as described in [83], [84]. When MS BLAST confirmed confidently the borderline protein identification by Mascot, the hit was considered as validated and was reported. To identify the orthologues of *Xenopus* proteins we used reciprocal BLAST searches with two candidate sequences - from *Xenopus* and the species to which belonged the hit (in most cases *Xenopus tropicalis*, *Homo sapiens* and *Mus musculus*).

A single function and/or localization was assigned to proteins using information in following databases: ExPASy Proteomics Server (http://www.expasy.org), GeneCards (http://www.genecards.org) and PubMed (http://www.ncbi.nlm.nih.gov/entrez/query.fcgi?db=PubMed).

### Antibodies and cDNAs

Anti-α-tubulin monoclonal antibodies were generously donated by L. Lafanechère. Full-length ORF cDNAs were obtained from “Deutsches Ressourcenzentrum für Genomforschung” (RZPD) and Kazusa clone collections.

### GFP-Tagged Proteins

cDNAs corresponding to the identified proteins were amplified by PCR using a high-fidelity thermostable DNA polymerase Phusion™ (Finnzymes) for ten cycles using manufacturer's instructions. Primers used for PCR are listed in Table S5. Amplified fragments were digested with appropriate restriction enzymes, gel-purified and ligated into an eukaryotic expression vector (pEGFP-C1, Clontech) or pHAT2 [85]. Recombinant proteins were produced in BL21 E.coli and purified using Talon™ resin (Clontech) according to manufacturer's instructions.

### Cell Transfection and Immunofluorescence Microscopy

*Xenopus* A6 [86] and XL177 [87] cells were grown at 20°C in 60% Leibowitz-15 medium with 10% FCS, 10 mM HEPES, pH 7.2, and antibiotics. HeLa [88] and HEK293 (American Type Culture Collection Number CRL-1573) cells were grown in DMEM supplemented with 10% FCS and antibiotics at 37°C. Plasmids were transfected into cells using calcium phosphate method [89] or Lipofectamine™ (Invitrogen) according to manufacturer's instructions. Cells were observed 24–48 hours after transfection in Zeiss Axiovert 200M inverted microscope using a standard Alexa488/FITC bandpass filter. Images were acquired using CoolSnap HQ (Photometrics) black and white camera driven by the Metamorph software (Universal Imaging). For siRNA-mediated knock-down, we transfected HeLa cells using Oligofectamine™ according to manufacturer's instructions. Prior to transfection cells were cultured overnight in serum and antibiotics-free OptiMEM (Gibco BRL). For the list of siRNA sequences see Table S6. To calculate the mitotic index we counted at least 7 microscopic fields (20x) containing between 560 and 2078 cells per siRNA-transfected sample.

### Network Building

The map of the microtubule proteome was initially compiled using PathwayStudio 4.0, a Windows PC-based application (Ariadne Genomics, Inc.). After compiling the Core map with all proteins binding directly to microtubules, we related all identified known proteins using information available in the Ariadne Genomics ResNet 3.0 database, followed by manual curation. In the resulting network, connections between proteins or proteins complexes were deliberately limited to the following relations: “binding”, “regulation” (positive or negative) and “phosphorylation” [90]. We created several proteins complexes, which were absent in the database (multi t-RNA synthetase complex, RNA-containing Kif5B-transported granules, HURP-complex and origin recognition complex). Entries listed in Table S1 represent identified *Xenopus* proteins (column “F”) and their human orthologues (column “G”). When the names of the human and *Xenopus* proteins were different, we provide in the Network the name of the *Xenopus* protein.

In order to provide open access to the network, we re-compiled it in Cytoscape [44], and further refined it manually. We added relevant “binding” links and specified references where a protein/microtubules or protein/protein association was previously described. For simplicity, the relationships of proteins which are in a complex are shown as « binding » this complex. All links were manually “curated” through links verification and cross-referencing in the NCBI PubMed database. The resulting network is available as Network S1 in the Supplemental Cytoscape session file.

In order to complement previously published protein interactions, experimental interaction evidence was collected from curated interaction databases. The following databases were downloaded: BIND [91], DIP [92], IntAct [93], HPRD [94], and MINT [95]. In addition, we used a literature-derived database, NetPro from Molecular Connections (http://www.molecularconnections.com/home/en/home/products/netPro).

Human orthologue entry is used for the search in the “GeneCards” database. Interactions were assigned to a protein pair in this study, if these proteins or their orthologues were described as interacting in a database. To this end, sequences of all proteins in the databases were compared with all proteins found in this study by a BLAST search using a 75% sequence identity cut-off. In rare instances, only low homology human orthologues of *Xenopus* proteins could be identified using reciprocal BLAST searches (e.g., *Xenopus* RNF36 and Xnf7 both match to human protein TRIM69, Acc. No. Q86WT6, with 42-43% of sequence identity). BLAST hits were checked manually and only correct orthologue assignments were retained. Confident interactions revealed low-throughput biochemical are shown in Network S2. Interactions revealed by high-throughput studies (and having lower confidence) are presented in Network S3. Table S4 lists all interactions.

## Supporting Information

Figure S1 Isolation of ATP-sensitive microtubule-bound proteins from extracts supplemented with AMPPNP or ATP. Gel shows proteins bound to microtubules from clarified (100,000 g) egg extracts and eluted by 20 mM ATP, as described in (Gache et al. 2007). Coomassie Blue-stained 6-18% gradient polyacrylamide SDS-electrophoresis gel. M - molecular weight markers (in kD). Lane 1- no nucleotides added; Lanes 2 - proteins bound to microtubules in the presence of 1.5mM AMP-PNP; Lane 3- proteins bound to microtubuless in the presence of 1.5mM AMP-PNP and 4 µM p50/dynamitin (Wittmann and Hyman 1999); Lane 4 -proteins bound in extract supplemented with 10 mM ATP without AMPPNP. Practically no protein bands are observed in the Lane 4, confirming that Lanes 1-3 contain proteins specifically attached to microtubules in the ATP-sensitive fashion. Major band at ∼50 kD corresponds to tubulin.
(1.24 MB TIF)

Figure S2 Proteins from ATP- and NaCl-elution fractions analyzed by a 2D-SDS-PAGE. Note that very little protein is visible above 100 kD. Staining with colloidal Coomassie Blue. Image reprinted from (Gache et al. 2007) with kind permission from Springer Science+Business Media, Purification and mass-spectrometry identification of microtubule binding proteins from Xenopus egg extracts. In: Zhou J, editor. Methods in Molecular Medicine: Microtubule Protocols. pp. 29-43, copyright 2007 Springer Science+Business Media.
(3.57 MB TIF)

Figure S3 Classification of identified proteins. Venn diagram in the centre shows the sizes of the ATP- and NaCl-eluted protein populations. Yellow color in Venn diagram represents the overlapping population of 93 proteins identified in both fractions. Pie charts show the classifications of identified proteins in the total assembly (left-hand upper corner) and in each fraction, including the overlapping fraction (right-hand lower corner). Note that each protein was assigned to only one category based on its most generally accepted function/localization.
(1.09 MB TIF)

Figure S4 Core map. MeMP proteins interacting directly with microtubules. Color code: MAPs are in blue, motors are in green, tubulin family members are in turquoise, protein complexes are in pink and the microtubule-actin binding protein MACF1 is in orange.
(2.07 MB TIF)

Figure S5 Localization of GFP-tagged candidate proteins in somatic cells. A. GFP-tagged candidate proteins observed live in interphase cells. Note midbody staining by GFP-Mgc81475. B. GFP-tagged candidate proteins observed live in mitotic cells. Mitotic state of the cells was confirmed by phase contrast microscopy (not shown). C. GFP-TSGA14 decorated interphase microtubules and the centrosome. D. GFP-LSM14A decorated interphase microtubules (“bundles”) and the “outer” midbody. Note that midbody staining of GFP-LSM14A shows a relatively long microtubule structure with a gap in the middle, while GFP-Mgc81475 forms a ring in the central part of the midbody (similar to Flemming body). GFP-tagged cDNAs were transfected into Xenopus (Mgc68500, Mgc80835, Mgc80361, Loc398535, Nif3l1bp1 and Mgc81475) and human (LSM14A, TSGA14 and Kiaa1799) cells. All images show representative phenotypes. Size bar - 10 µm.
(10.24 MB TIF)

Figure S6 Mitotic index in HeLa cells treated with siRNA against the human orthologue of Mgc81475. Mitotic index (MI) was calculated as the percentage of phosphohistone H3-positive cells. Mean MI shown on the left was calculated for ten huMgc81475-unrelated siRNAs listed in the Table S6, (all siRNAs excepting #3 and 4). At least seven microscopic fields (at magnification 20x) were evaluated per sample with total number of cells per sample between 560 and 2078. Error bar - standard deviation of the mean.
(0.72 MB TIF)

Figure S7 Mitotic spindle proteome versus meiotic microtubule proteome. Venn diagram showing the overlap between MSP (in red; (Sauer et al. 2005)) and MeMP (in green; this study). Yellow color shows the size of the population common to both protein lists.
(1.11 MB TIF)

Figure S8 GFP-TSGA14 localization. A. Live cell imaging showing GFP-TSGA14 decorating microtubules. B. Live cell imaging showing a centrosome-like structure in a cell expressing low amounts of GFP-TSGA14. C. Immunolocalization of gamma-tubulin (Sigma, GTU-88) in a HeLa cell expressing low amounts of GFP-TSGA14. Fixation: 4% PFA.
(2.10 MB TIF)

Table S1 Summary of identified proteins. ATP- and NaCl-elution fractions and combined list with 318 identifications. Different color code was used for such categories as: MAPs, Motors, Vesicular traffic, Signaling, Protein synthesis and folding, Protein degradation, Metabolism, Cytoskeletal proteins, Actin and intermediate filaments binding proteins, Centrosomal proteins, Cell cycle, RNA transcription/processing/structure, DNA replication/reparation/structure and Others. Column “A” indicates in which fraction was identified a given protein. Column “F” and “G” contain the names of Xenopus and human proteins respectively. Sheets 2, 3 and 4 contain the lists of proteins identified in the ATP, NaCl fractions and 2D gels, respectively.
(0.41 MB XLS)

Table S2 Sheet 1. List of “MAPs” and motors found in MSP (Sauer et al. 2005) and MeMP (this study). Yellow, green and blue areas show proteins common to both proteomes, MSP and MeMP, respectively. Note that certain known spindle components are listed in the current Table as “MAPs” following the classification of (Sauer et al. 2005), while we placed them into other categories in Supplementary Table 1 and Supplementary Figure 3. Sheet 2. List of eleven known MAPs absent from the MSP (Sauer et al. 2005) with their localizations.
(0.04 MB XLS)

Table S3 List of 90 proteins common to MSP and MeMP. Note that only five proteins from the list do not have a known connectivity with other proteins from MeMP and are thus absent from Figures 2 and 5.
(0.40 MB XLS)

Table S4 List of the meiotic microtubule protein interaction network entities (proteins shown in Figure 2). Column “I” contains PubMed references pertaining to links shown in Figure 2.
(0.13 MB XLS)

Table S5 Sequences of PCR primers used to amplify cDNAs coding for candidate proteins with expected PCR fragment sizes and protein masses.
(0.04 MB DOC)

Table S6 Sequences of siRNAs used for HeLa cells transfection.
(0.04 MB DOC)

## References

[1] Castedo M, Kroemer G (2004). [Mitotic catastrophe: a special case of apoptosis].. J Soc Biol.

[2] Shi Q, King RW (2005). Chromosome nondisjunction yields tetraploid rather than aneuploid cells in human cell lines.. Nature.

[3] Fujiwara T, Bandi M, Nitta M, Ivanova EV, Bronson RT (2005). Cytokinesis failure generating tetraploids promotes tumorigenesis in p53-null cells.. Nature.

[4] Nicolaidis P, Petersen MB (1998). Origin and mechanisms of non-disjunction in human autosomal trisomies.. Hum Reprod.

[5] Hassold T, Hunt P (2001). To err (meiotically) is human: the genesis of human aneuploidy.. Nat Rev Genet.

[6] Pellestor F, Andreo B, Arnal F, Humeau C, Demaille J (2003). Maternal aging and chromosomal abnormalities: new data drawn from in vitro unfertilized human oocytes.. Hum Genet.

[7] Hitzler JK, Zipursky A (2005). Origins of leukaemia in children with Down syndrome.. Nat Rev Cancer.

[8] Lamb NE, Feingold E, Savage A, Avramopoulos D, Freeman S (1997). Characterization of susceptible chiasma configurations that increase the risk for maternal nondisjunction of chromosome 21.. Hum Mol Genet.

[9] Cukurcam S, Sun F, Betzendahl I, Adler ID, Eichenlaub-Ritter U (2004). Trichlorfon predisposes to aneuploidy and interferes with spindle formation in in vitro maturing mouse oocytes.. Mutat Res.

[10] Battaglia DE, Goodwin P, Klein NA, Soules MR (1996). Influence of maternal age on meiotic spindle assembly in oocytes from naturally cycling women.. Hum Reprod.

[11] Wang WH, Sun QY (2006). Meiotic spindle, spindle checkpoint and embryonic aneuploidy.. Front Biosci.

[12] Mailhes JB, Mastromatteo C, Fuseler JW (2004). Transient exposure to the Eg5 kinesin inhibitor monastrol leads to syntelic orientation of chromosomes and aneuploidy in mouse oocytes.. Mutat Res.

[13] Simerly C, Dominko T, Navara C, Payne C, Capuano S (2003). Molecular correlates of primate nuclear transfer failures.. Science.

[14] Brunet S, Maria AS, Guillaud P, Dujardin D, Kubiak JZ (1999). Kinetochore fibers are not involved in the formation of the first meiotic spindle in mouse oocytes, but control the exit from the first meiotic M phase.. J Cell Biol.

[15] Dumont J, Petri S, Pellegrin F, Terret ME, Bohnsack MT (2007). A centriole- and RanGTP-independent spindle assembly pathway in meiosis I of vertebrate oocytes.. J Cell Biol.

[16] Wittmann T, Hyman A, Desai A (2001). The spindle: a dynamic assembly of microtubules and motors.. Nat Cell Biol.

[17] Sloboda RD, Rudolph SA, Rosenbaum JL, Greengard P (1975). Cyclic AMP-dependent endogenous phosphorylation of a microtubule-associated protein.. Proc Natl Acad Sci U S A.

[18] Weingarten MD, Lockwood AH, Hwo SY, Kirschner MW (1975). A protein factor essential for microtubule assembly.. Proc Natl Acad Sci U S A.

[19] Schliwa M, Woehlke G (2003). Molecular motors.. Nature.

[20] Desai A, Verma S, Mitchison TJ, Walczak CE (1999). Kin I kinesins are microtubule-destabilizing enzymes.. Cell.

[21] Morejohn LC (1994). Microtubule Binding Proteins Are Not Necessarily Microtubule-Associated Proteins.. Plant Cell.

[22] Cassimeris L, Spittle C (2001). Regulation of microtubule-associated proteins.. Int Rev Cytol.

[23] Hirokawa N (1994). Microtubule organization and dynamics dependent on microtubule-associated proteins.. Curr Opin Cell Biol.

[24] Ookata K, Hisanaga S, Bulinski JC, Murofushi H, Aizawa H (1995). Cyclin B interaction with microtubule-associated protein 4 (MAP4) targets p34cdc2 kinase to microtubules and is a potential regulator of M-phase microtubule dynamics.. J Cell Biol.

[25] Hirokawa N, Noda Y, Okada Y (1998). Kinesin and dynein superfamily proteins in organelle transport and cell division.. Curr Opin Cell Biol.

[26] Liska AJ, Popov AV, Sunyaev S, Coughlin P, Habermann B (2004). Homology-based functional proteomics by mass spectrometry: application to the *Xenopus* microtubule-associated proteome.. Proteomics.

[27] Andersen JS, Wilkinson CJ, Mayor T, Mortensen P, Nigg EA (2003). Proteomic characterization of the human centrosome by protein correlation profiling.. Nature.

[28] Sauer G, Korner R, Hanisch A, Ries A, Nigg EA (2005). Proteome analysis of the human mitotic spindle.. Mol Cell Proteomics.

[29] Skop AR, Liu H, Yates J, Meyer BJ, Heald R (2004). Dissection of the mammalian midbody proteome reveals conserved cytokinesis mechanisms.. Science.

[30] Mack GJ, Compton DA (2001). Analysis of mitotic microtubule-associated proteins using mass spectrometry identifies astrin, a spindle-associated protein.. Proc Natl Acad Sci U S A.

[31] Lohka MJ, Masui Y (1983). Formation in vitro of sperm pronuclei and mitotic chromosomes induced by amphibian ooplasmic components.. Science.

[32] Newport J, Kirschner M (1982). A major developmental transition in early *Xenopus* embryos: I. characterization and timing of cellular changes at the midblastula stage.. Cell.

[33] Waridel P, Frank A, Thomas H, Surendranath V, Sunyaev S (2007). Sequence Similarity-Driven Proteomics in Organisms with Unknown Genomes by LC-MS/MS and Automated De Novo Sequencing.. Proteomics.

[34] Gache V, Waridel P, Luche S, Shevchenko A, Popov AV,  Zhou J (2007). Purification and mass-spectrometry identification of microtubule binding proteins from *Xenopus* egg extracts.. Methods in Molecular Medicine: Microtubule Protocols.

[35] Ems-McClung SC, Zheng Y, Walczak CE (2004). Importin alpha/beta and Ran-GTP regulate XCTK2 microtubule binding through a bipartite nuclear localization signal.. Mol Biol Cell.

[36] Lajoie-Mazenc I, Tollon Y, Detraves C, Julian M, Moisand A (1994). Recruitment of antigenic gamma-tubulin during mitosis in animal cells: presence of gamma-tubulin in the mitotic spindle.. J Cell Sci.

[37] Wilde A, Zheng Y (1999). Stimulation of microtubule aster formation and spindle assembly by the small GTPase Ran.. Science.

[38] Luders J, Patel UK, Stearns T (2006). GCP-WD is a gamma-tubulin targeting factor required for centrosomal and chromatin-mediated microtubule nucleation.. Nat Cell Biol.

[39] Desai A, Murray A, Mitchison TJ, Walczak CE (1999). The use of *Xenopus* egg extracts to study mitotic spindle assembly and function in vitro.. Methods Cell Biol.

[40] Lenart P, Bacher CP, Daigle N, Hand AR, Eils R (2005). A contractile nuclear actin network drives chromosome congression in oocytes.. Nature.

[41] Weber KL, Sokac AM, Berg JS, Cheney RE, Bement WM (2004). A microtubule-binding myosin required for nuclear anchoring and spindle assembly.. Nature.

[42] Woolner S, O'Brien LL, Wiese C, Bement WM (2008). Myosin-10 and actin filaments are essential for mitotic spindle function.. J Cell Biol.

[43] Merdes A, Ramyar K, Vechio JD, Cleveland DW (1996). A complex of NuMA and cytoplasmic dynein is essential for mitotic spindle assembly.. Cell.

[44] Shannon P, Markiel A, Ozier O, Baliga NS, Wang JT (2003). Cytoscape: a software environment for integrated models of biomolecular interaction networks.. Genome Res.

[45] Liu J, Bang AG, Kintner C, Orth AP, Chanda SK (2005). Identification of the Wnt signaling activator leucine-rich repeat in Flightless interaction protein 2 by a genome-wide functional analysis.. Proc Natl Acad Sci U S A.

[46] Spike CA, Shaw JE, Herman RK (2001). Analysis of smu-1, a gene that regulates the alternative splicing of unc-52 pre-mRNA in Caenorhabditis elegans.. Mol Cell Biol.

[47] Gassmann R, Henzing A, Earnshaw W (2005). Novel components of human mitotic chromosomes identified by proteomic analysis of the chromosome scaffold fraction.. Chromosoma.

[48] Suprenant KA, Dean K, McKee J, Hake S (1993). EMAP, an echinoderm microtubule-associated protein found in microtubule-ribosome complexes.. J Cell Sci.

[49] Fields S, Song O (1989). A novel genetic system to detect protein-protein interactions.. Nature.

[50] Koffa MD, Casanova CM, Santarella R, Kocher T, Wilm M (2006). HURP is part of a Ran-dependent complex involved in spindle formation.. Curr Biol.

[51] Akhmanova A, Hoogenraad CC (2005). Microtubule plus-end-tracking proteins: mechanisms and functions.. Curr Opin Cell Biol.

[52] Eichenmuller B, Kedersha N, Solovyeva E, Everley P, Lang J (2003). Vaults bind directly to microtubules via their caps and not their barrels.. Cell Motil Cytoskeleton.

[53] Wang S, Liu Y, Adamson CL, Valdez G, Guo W (2004). The mammalian exocyst, a complex required for exocytosis, inhibits tubulin polymerization.. J Biol Chem.

[54] Walenta JH, Didier AJ, Liu X, Kramer H (2001). The Golgi-associated hook3 protein is a member of a novel family of microtubule-binding proteins.. J Cell Biol.

[55] Kanai Y, Dohmae N, Hirokawa N (2004). Kinesin transports RNA: isolation and characterization of an RNA-transporting granule.. Neuron.

[56] Pestova TV, Kolupaeva VG, Lomakin IB, Pilipenko EV, Shatsky IN (2001). Molecular mechanisms of translation initiation in eukaryotes.. Proc Natl Acad Sci U S A.

[57] Yoshida S, Simbulan CM (1994). Interaction of poly(ADP-ribose)polymerase with DNA polymerase alpha.. Mol Cell Biochem.

[58] Merz C, Urlaub H, Will CL, Luhrmann R (2007). Protein composition of human mRNPs spliced in vitro and differential requirements for mRNP protein recruitment.. Rna.

[59] Eulalio A, Behm-Ansmant I, Izaurralde E (2007). P bodies: at the crossroads of post-transcriptional pathways.. Nat Rev Mol Cell Biol.

[60] Blower MD, Feric E, Weis K, Heald R (2007). Genome-wide analysis demonstrates conserved localization of messenger RNAs to mitotic microtubules.. J Cell Biol.

[61] Casaletto JB, Nutt LK, Wu Q, Moore JD, Etkin LD (2005). Inhibition of the anaphase-promoting complex by the Xnf7 ubiquitin ligase.. J Cell Biol.

[62] Maresca TJ, Niederstrasser H, Weis K, Heald R (2005). Xnf7 contributes to spindle integrity through its microtubule-bundling activity.. Curr Biol.

[63] Chen XW, Inoue M, Hsu SC, Saltiel AR (2006). RalA-exocyst-dependent recycling endosome trafficking is required for the completion of cytokinesis.. J Biol Chem.

[64] Rual JF, Venkatesan K, Hao T, Hirozane-Kishikawa T, Dricot A (2005). Towards a proteome-scale map of the human protein-protein interaction network.. Nature.

[65] Cha B, Cassimeris L, Gard DL (1999). XMAP230 is required for normal spindle assembly in vivo and in vitro.. J Cell Sci.

[66] Simpson JC, Wellenreuther R, Poustka A, Pepperkok R, Wiemann S (2000). Systematic subcellular localization of novel proteins identified by large-scale cDNA sequencing.. EMBO Rep.

[67] Simpson JC, Pepperkok R (2006). The subcellular localization of the mammalian proteome comes a fraction closer.. Genome Biol.

[68] Huang L, Grammatikakis N, Yoneda M, Banerjee SD, Toole BP (2000). Molecular characterization of a novel intracellular hyaluronan-binding protein.. J Biol Chem.

[69] Groen AC, Cameron LA, Coughlin M, Miyamoto DT, Mitchison TJ (2004). XRHAMM functions in ran-dependent microtubule nucleation and pole formation during anastral spindle assembly.. Curr Biol.

[70] Rehwinkel J, Herold A, Gari K, Kocher T, Rode M (2004). Genome-wide analysis of mRNAs regulated by the THO complex in Drosophila melanogaster.. Nat Struct Mol Biol.

[71] Popov AV, Pozniakovsky A, Arnal I, Antony C, Ashford AJ (2001). XMAP215 regulates microtubule dynamics through two distinct domains.. Embo J.

[72] Tanaka KJ, Ogawa K, Takagi M, Imamoto N, Matsumoto K (2006). RAP55, a cytoplasmic mRNP component, represses translation in *Xenopus* oocytes.. J Biol Chem.

[73] Groisman I, Huang YS, Mendez R, Cao Q, Theurkauf W (2000). CPEB, maskin, and cyclin B1 mRNA at the mitotic apparatus: implications for local translational control of cell division.. Cell.

[74] Scholey JM, Brust-Mascher I, Mogilner A (2003). Cell division.. Nature.

[75] Khodjakov A, Cole RW, Oakley BR, Rieder CL (2000). Centrosome-independent mitotic spindle formation in vertebrates.. Curr Biol.

[76] Castoldi M, Popov AV (2003). Purification of brain tubulin through two cycles of polymerization-depolymerization in a high-molarity buffer.. Protein Expr Purif.

[77] Murray AW (1991). Cell cycle extracts.. Methods Cell Biol.

[78] Hyman A, Drechsel D, Kellogg D, Salser S, Sawin K (1991). Preparation of modified tubulins.. Methods Enzymol.

[79] Sawin KE, Mitchison TJ (1991). Mitotic spindle assembly by two different pathways in vitro.. J Cell Biol.

[80] Shevchenko A, Wilm M, Vorm O, Mann M (1996). Mass spectrometric sequencing of proteins from silver-stained polyacrylamide gels.. Anal Chem.

[81] Frank A, Pevzner P (2005). PepNovo: de novo peptide sequencing via probabilistic network modeling.. Anal Chem.

[82] Shevchenko A, Sunyaev S, Loboda A, Bork P, Ens W (2001). Charting the proteomes of organisms with unsequenced genomes by MALDI-quadrupole time-of-flight mass spectrometry and BLAST homology searching.. Anal Chem.

[83] Shevchenko A, Sunyaev S, Liska A, Bork P (2003). Nanoelectrospray tandem mass spectrometry and sequence similarity searching for identification of proteins from organisms with unknown genomes.. Methods Mol Biol.

[84] Habermann B, Oegema J, Sunyaev S, Shevchenko A (2004). The power and the limitations of cross-species protein identification by mass spectrometry-driven sequence similarity searches.. Mol Cell Proteomics.

[85] Peranen J, Rikkonen M, Hyvonen M, Kaariainen L (1996). T7 vectors with modified T7lac promoter for expression of proteins in Escherichia coli.. Anal Biochem.

[86] Rafferty KA, Mizell M (1969). Mass culture of amphibia cells: Methods and observations concerning stability of cell type.. Biology of Amphibian Tumors.

[87] Miller L, Daniel JC (1977). Comparison of in vivo and in vitro ribosomal RNA synthesis in nucleolar mutants of *Xenopus* laevis.. In Vitro.

[88] Scherer WF, Syverton JT, Gey GO (1953). Studies on the propagation in vitro of poliomyelitis viruses. IV. Viral multiplication in a stable strain of human malignant epithelial cells (strain HeLa) derived from an epidermoid carcinoma of the cervix.. J Exp Med.

[89] Bonifacino JS, Dasso M, Harford JB, Lippincott-Schwartz J, Yamada KM (2006).

[90] Nikitin A, Egorov S, Daraselia N, Mazo I (2003). Pathway studio–the analysis and navigation of molecular networks.. Bioinformatics.

[91] Bader GD, Betel D, Hogue CW (2003). BIND: the Biomolecular Interaction Network Database.. Nucleic Acids Res.

[92] Salwinski L, Miller CS, Smith AJ, Pettit FK, Bowie JU (2004). The Database of Interacting Proteins: 2004 update.. Nucleic Acids Res.

[93] Hermjakob H, Montecchi-Palazzi L, Lewington C, Mudali S, Kerrien S (2004). IntAct: an open source molecular interaction database.. Nucleic Acids Res.

[94] Mishra GR, Suresh M, Kumaran K, Kannabiran N, Suresh S (2006). Human protein reference database–2006 update.. Nucleic Acids Res.

[95] Zanzoni A, Montecchi-Palazzi L, Quondam M, Ausiello G, Helmer-Citterich M (2002). MINT: a Molecular INTeraction database.. FEBS Lett.

