# Supplementary figures and images for: Xenopus Meiotic Microtubule-Associated Interactome
Source: PLoS One. 2010 Feb 17;5(2):e9248. doi: 10.1371/journal.pone.0009248 (PMC2822853; doi:10.1371/journal.pone.0009248)

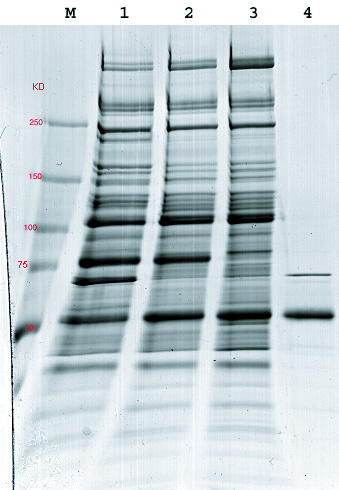

Supplement: Figure S1 — Isolation of ATP-sensitive microtubule-bound proteins from extracts supplemented with AMPPNP or ATP. Gel shows proteins bound to microtubules from clarified (100,000 g) egg extracts and eluted by 20 mM ATP, as described in (Gache et al. 2007). Coomassie Blue-stained 6-18% gradient polyacrylamide SDS-electrophoresis gel. M - molecular weight markers (in kD). Lane 1- no nucleotides added; Lanes 2 - proteins bound to microtubules in the presence of 1.5mM AMP-PNP; Lane 3- proteins bound to microtubuless in the presence of 1.5mM AMP-PNP and 4 µM p50/dynamitin (Wittmann and Hyman 1999); Lane 4 -proteins bound in extract supplemented with 10 mM ATP without AMPPNP. Practically no protein bands are observed in the Lane 4, confirming that Lanes 1-3 contain proteins specifically attached to microtubules in the ATP-sensitive fashion. Major band at ∼50 kD corresponds to tubulin. (1.24 MB TIF) [file pone.0009248.s001.tif]

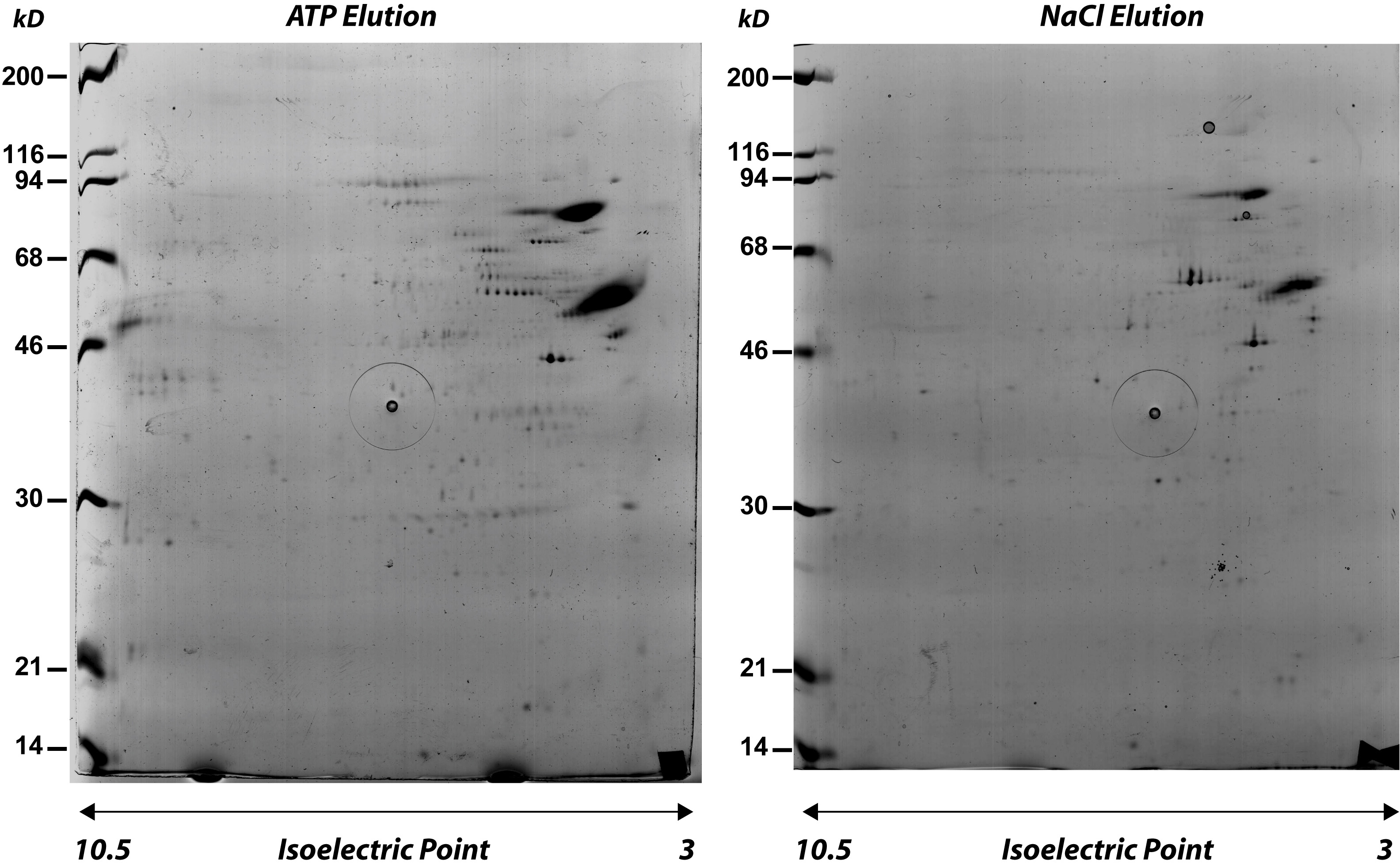

Supplement: Figure S2 — Proteins from ATP- and NaCl-elution fractions analyzed by a 2D-SDS-PAGE. Note that very little protein is visible above 100 kD. Staining with colloidal Coomassie Blue. Image reprinted from (Gache et al. 2007) with kind permission from Springer Science+Business Media, Purification and mass-spectrometry identification of microtubule binding proteins from Xenopus egg extracts. In: Zhou J, editor. Methods in Molecular Medicine: Microtubule Protocols. pp. 29-43, copyright 2007 Springer Science+Business Media. (3.57 MB TIF) [file pone.0009248.s002.tif]

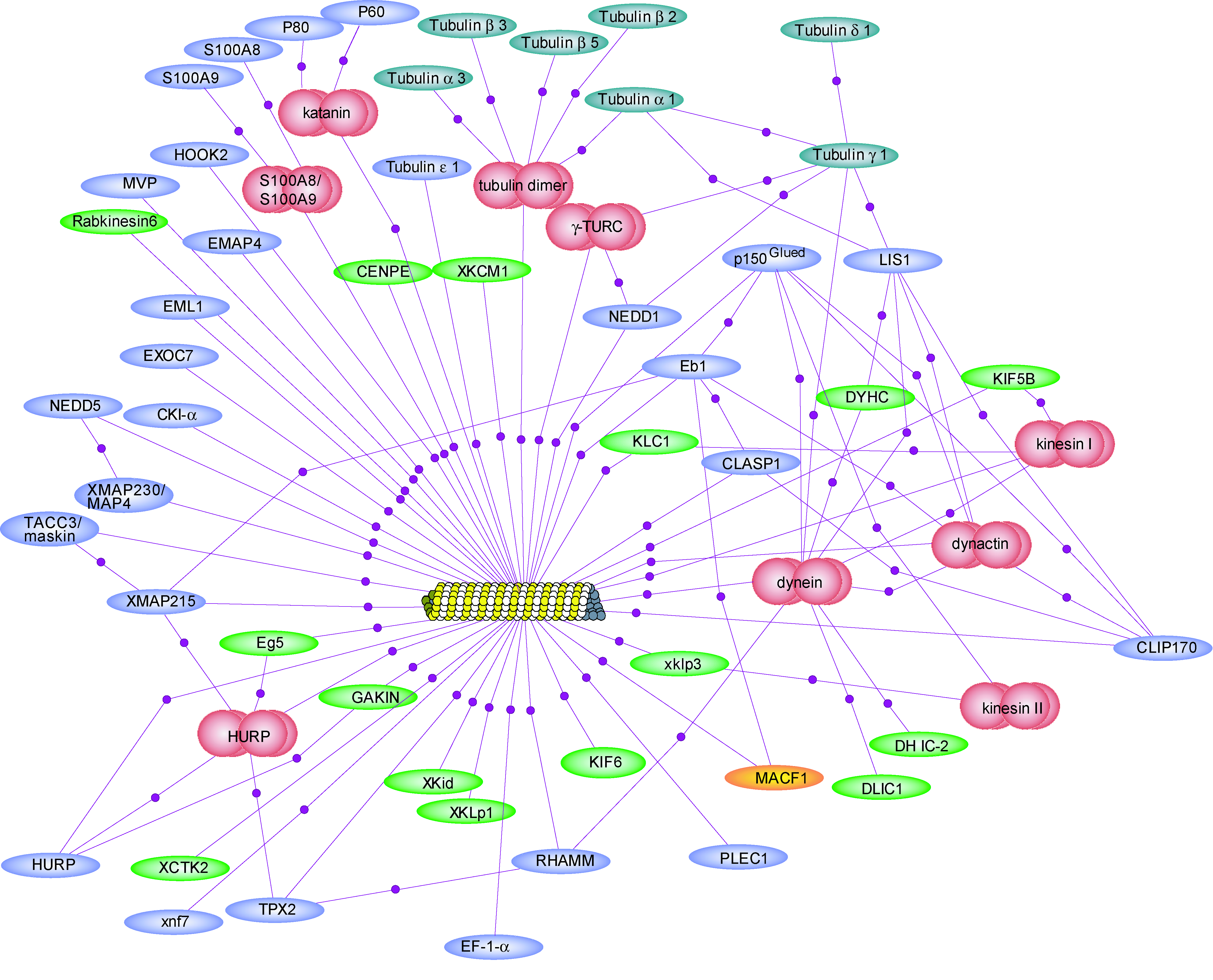

Supplement: Figure S4 — Core map. MeMP proteins interacting directly with microtubules. Color code: MAPs are in blue, motors are in green, tubulin family members are in turquoise, protein complexes are in pink and the microtubule-actin binding protein MACF1 is in orange. (2.07 MB TIF) [file pone.0009248.s004.tif]

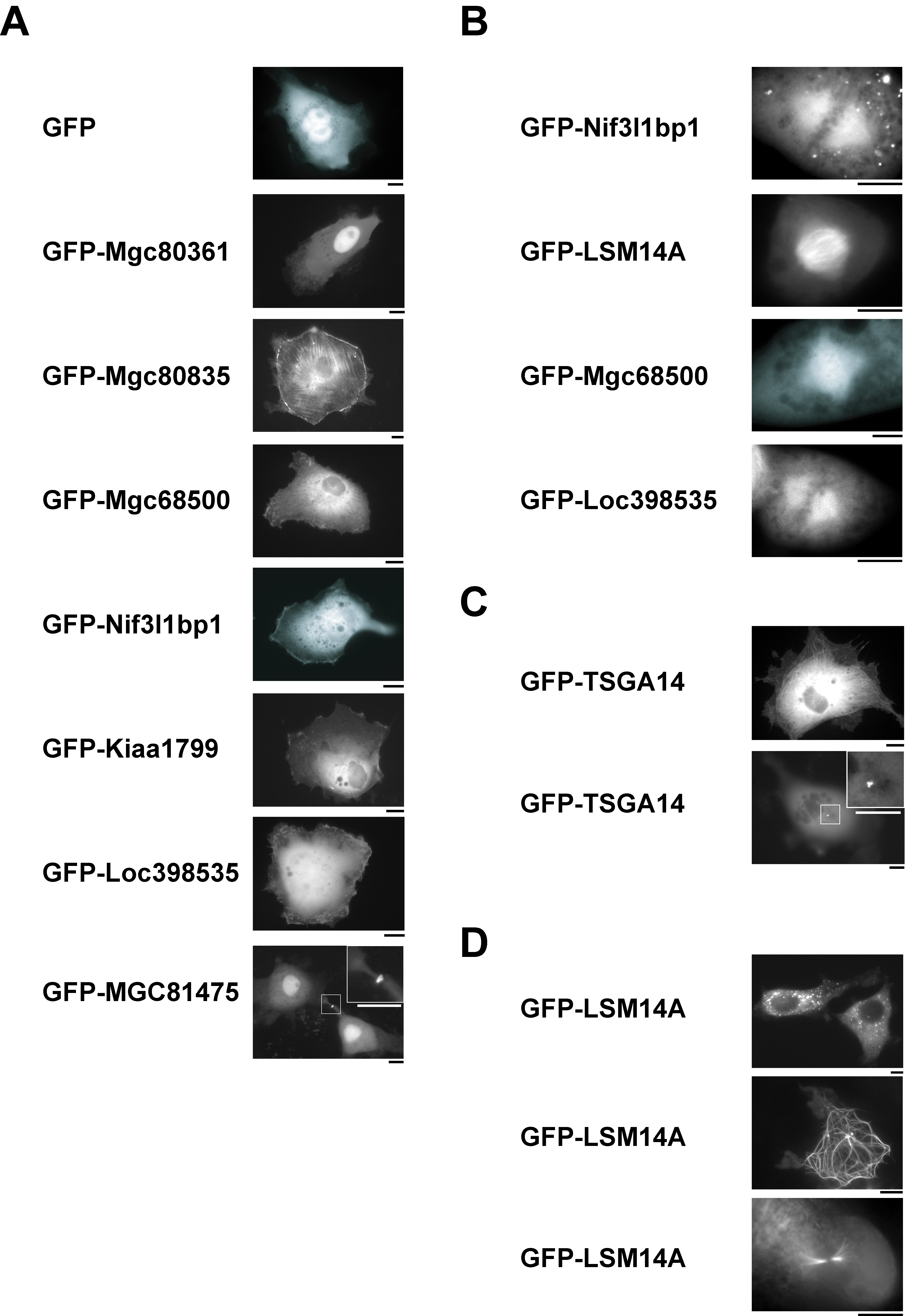

Supplement: Figure S5 — Localization of GFP-tagged candidate proteins in somatic cells. A. GFP-tagged candidate proteins observed live in interphase cells. Note midbody staining by GFP-Mgc81475. B. GFP-tagged candidate proteins observed live in mitotic cells. Mitotic state of the cells was confirmed by phase contrast microscopy (not shown). C. GFP-TSGA14 decorated interphase microtubules and the centrosome. D. GFP-LSM14A decorated interphase microtubules (“bundles”) and the “outer” midbody. Note that midbody staining of GFP-LSM14A shows a relatively long microtubule structure with a gap in the middle, while GFP-Mgc81475 forms a ring in the central part of the midbody (similar to Flemming body). GFP-tagged cDNAs were transfected into Xenopus (Mgc68500, Mgc80835, Mgc80361, Loc398535, Nif3l1bp1 and Mgc81475) and human (LSM14A, TSGA14 and Kiaa1799) cells. All images show representative phenotypes. Size bar - 10 µm. (10.24 MB TIF) [file pone.0009248.s005.tif]

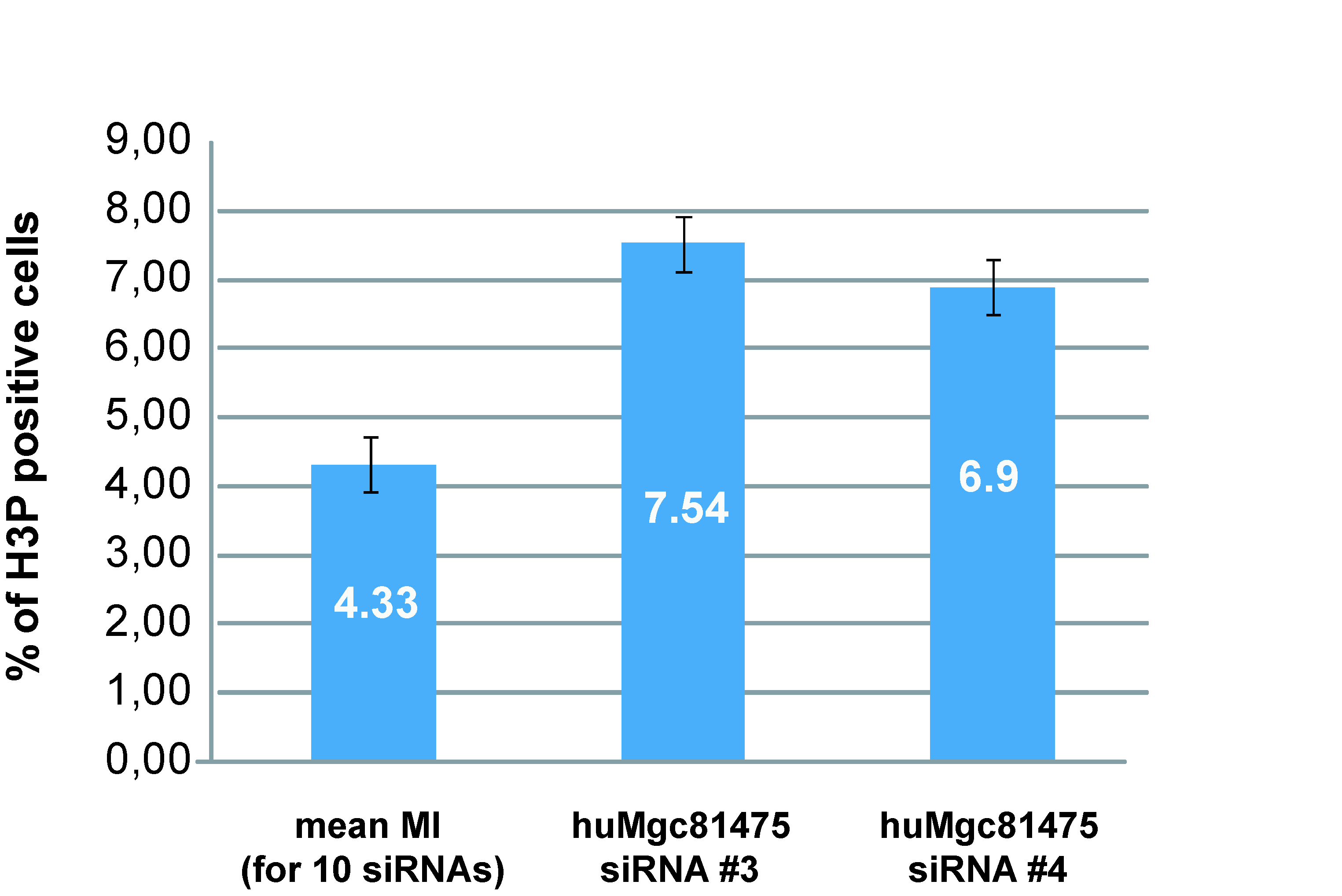

Supplement: Figure S6 — Mitotic index in HeLa cells treated with siRNA against the human orthologue of Mgc81475. Mitotic index (MI) was calculated as the percentage of phosphohistone H3-positive cells. Mean MI shown on the left was calculated for ten huMgc81475-unrelated siRNAs listed in the Table S6, (all siRNAs excepting #3 and 4). At least seven microscopic fields (at magnification 20x) were evaluated per sample with total number of cells per sample between 560 and 2078. Error bar - standard deviation of the mean. (0.72 MB TIF) [file pone.0009248.s006.tif]

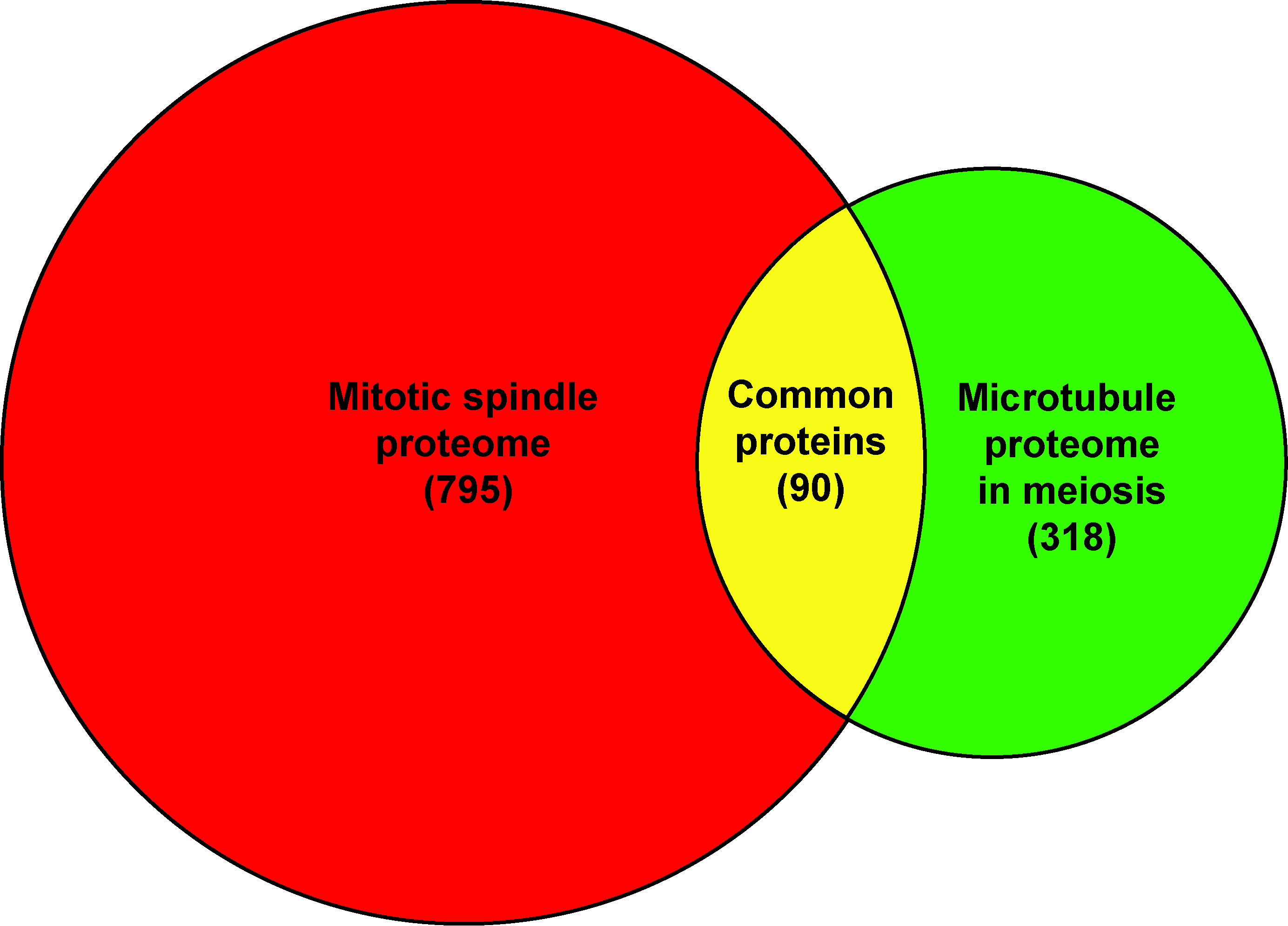

Supplement: Figure S7 — Mitotic spindle proteome versus meiotic microtubule proteome. Venn diagram showing the overlap between MSP (in red; (Sauer et al. 2005)) and MeMP (in green; this study). Yellow color shows the size of the population common to both protein lists. (1.11 MB TIF) [file pone.0009248.s007.tif]

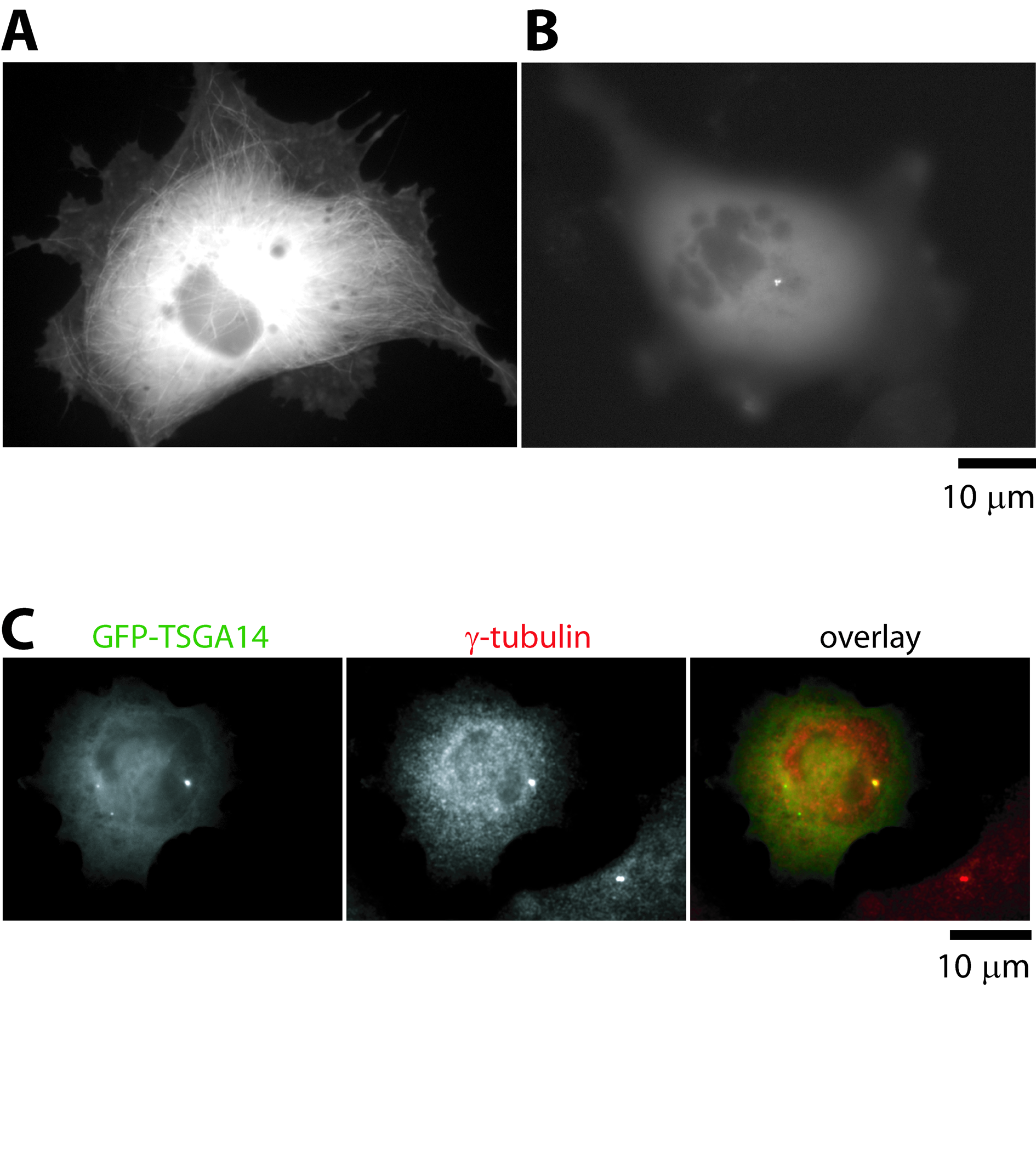

Supplement: Figure S8 — GFP-TSGA14 localization. A. Live cell imaging showing GFP-TSGA14 decorating microtubules. B. Live cell imaging showing a centrosome-like structure in a cell expressing low amounts of GFP-TSGA14. C. Immunolocalization of gamma-tubulin (Sigma, GTU-88) in a HeLa cell expressing low amounts of GFP-TSGA14. Fixation: 4% PFA. (2.10 MB TIF) [file pone.0009248.s008.tif]
