# Supplementary material for: Xenopus Meiotic Microtubule-Associated Interactome
Source: PLoS One. 2010 Feb 17;5(2):e9248. doi: 10.1371/journal.pone.0009248 (PMC2822853; doi:10.1371/journal.pone.0009248)
Supplement: Table S5 — Sequences of PCR primers used to amplify cDNAs coding for candidate proteins with expected PCR fragment sizes and protein masses. (0.04 MB DOC) [file pone.0009248.s013.doc]

| No | Clone/ ExPASy Acc. No | Forward Primer | Reverse Primer | Expected size  DNA fragment (bp) | Expected [MW](http://www.expasy.org/sprot/userman.html#SQ_line)  protein  without GFP (D) |
| --- | --- | --- | --- | --- | --- |
| 1 | Nif3l1bp1-prov/ Q7SZ78 | 5’-AGCTCGAGATATGGGAGCCGTCAC-3’ | 5’-GAGGATCCTGGATTTTGAGTTTCC-3’ | 606 | 23371 |
| 2 | Mgc 80835/  Q6GNW0 | 5’-AGCTCGAGATATGGGGACTCCTGG-3’ | 5’-ACCCGGGAACTGCTGAGACAGAAG-3’ | 1218 | 46179 |
| 3 | Mgc 68500/  Q6PB22 | 5’-AGCTCGAGATATGCGATTGGATACAAT-3’ | 5’-GAGGATCCAGCCAAAGCAGGGAA-3’ | 1215 | 45528 |
| 4 | Loc 398535/  Q6GQL3 | 5’-AGCTCGAGATATGTCGGAGAAGTCA-3’ | 5’-GAGGATCCCCAGCTTTCTGTTTTC-3’ | 1077 | 38696 |
| 5 | Mgc 80361/  Q6AXA1 | 5’-AGCTCGAGATATGGAGAAGCAAACG-3’ | 5’-GAGGATCCCTTCTTCCTCCTTTTA-3’ | 1158 | 42758 |
| 6 | Mgc81475/ Q6NRT3 | 5’ AGCTCGAGATATGTCTATCGAAATTGA-3’ | 5’ GAGGATCCCGGCTTCCACAGTTT-3’ | 1542 | 57623 |
| 7 | TSGA14/  Q9BYV8 | 5’-AGCTCGAGATATGTCCCTCCGGAG-3’ | 5’-GAGGATCCCTTCCAGGGTTTGC-3’ | 906 | 41368 |
| 8 | Kiaa1799/  Q96B95 | 5’-TATGTCGACATGGCGATCAGTCCA-3’ | 5’-GAGGATCC AGGCATTGTAGATTTG-3’ | 1821 | 71935 |
| 9 | LSM14A/  Q8ND56 | 5’-TATGTCGACATGAGCGGGGGCA-3’ | 5’-GAGGATCCTGCAGCAACTTTGTTG-3’ | 1392 | 50530 |
