# Supplementary material for: Xenopus Meiotic Microtubule-Associated Interactome
Source: PLoS One. 2010 Feb 17;5(2):e9248. doi: 10.1371/journal.pone.0009248 (PMC2822853; doi:10.1371/journal.pone.0009248)
Supplement: Table S6 — Sequences of siRNAs used for HeLa cells transfection. (0.04 MB DOC) [file pone.0009248.s014.doc]

**Supplemental Table 6**

| No | Xenopus protein/  Human  analogue | Sequence |
| --- | --- | --- |
| 1 | RAP55A/  LSM14A | 5’-AGU AGU GCG GUU GGU UCU G dTdT-3’  5’-CAG AAC CAA CCG CAC UAC U dTdT-3’ |
| 2 | RAP55A/  LSM14A | 5’-GCU GAA GAA AGA AGA UUA A dTdT-3’  5’-UUA AUC UUC UUU CUU CAG C dTdT-3’ |
| 3 | Mgc81475/ hSMU1 | 5’-CGG UGG UCA UCA UGA ACA U dTdT-3’  5’-AUG UUC AUG AUG ACC ACC G dTdT-3’ |
| 4 | Mgc81475/ hSMU1 | 5’-GAU UCA GAG UGG ACA AUG U dTdT-3’  5’-ACA UUG UCC ACU CUG AAU C dTdT-3’ |
| 5 | Loc398535/  BAT2DP1 | 5’-GGC AAU GAU CCU AAU GUA A dTdT-3’  5’-UUA CAU UAG GAU CAU UGC C dTdT-3’ |
| 6 | Loc398535/  BAT2DP1 | 5’- GUA GCA CCU GCU CCC AAA U dTdT-3’  5’- AUU UGG GAG CAG GUG CUA C dTdT-3’ |
| 7 | Nif3l1bp1/  Thoc7 | 5’-GAG CAU CUU UCA CAC AUU A dTdT-3’  5’-UAA UGU GUG AAA GAU GCU C dTdT-3’ |
| 8 | Nif3l1bp1/  Thoc7 | 5’-GAC AGG CAU GAG ACA UUA A dTdT-3’  5’-UUA AUG UCU CAU GCC UGU C dTdT-3’ |
| 9 | TSGA14/  Cep41 | 5’-CUG GUA ACA GUA UGA CUA A dTdT-3’  5’-UUA GUC AUA CUG UUA CCA G dTdT-3’ |
| 10 | TSGA14/  Cep41 | 5’-CCA GCA GUG CCA CAU UGU U dTdT-3’  5’-AAC AAU GUG GCA CUG CUG G dTdT-3’ |
| 11 | Mgc68500/  IHABP4 | 5’-CCA GAC CAA AGC CUG AGU U dTdT-3’  5’-AAC UCA GGC UUU GGU CUG G dTdT-3’ |
| 12 | Mgc68500/  IHABP4 | 5’-CAU CCC AGC UGG AGA UUA A dTdT-3’  5’-UUA AUC UCC AGC UGG GAU G dTdT-3’ |

Sequences of siRNAs used for HeLa cells transfection
